# Preparation and Property Characterization of Sm_2_EuSbO_7_/ZnBiSbO_5_ Heterojunction Photocatalyst for Photodegradation of Parathion Methyl under Visible Light Irradiation

**DOI:** 10.3390/molecules28237722

**Published:** 2023-11-22

**Authors:** Jingfei Luan, Liang Hao, Ye Yao, Yichun Wang, Guangmin Yang, Jun Li

**Affiliations:** 1 School of Physics, Changchun Normal University, Changchun 130032, China; hliang0725@163.com (L.H.); yaoye1109@mails.jlu.edu.cn (Y.Y.); yichun2000@126.com (Y.W.); yangguangmin@ccsfu.edu.cn (G.Y.); 18043182231@163.com (J.L.); 2 State Key Laboratory of Pollution Control and Resource Reuse, School of the Environment, Nanjing University, Nanjing 210093, China

**Keywords:** Sm_2_EuSbO_7_, Sm_2_EuSbO_7_/ZnBiSbO_5_ heterojunction photocatalyst, parathion methyl, visible light irradiation, photocatalytic activity, degradation pathway, degradation mechanism

## Abstract

An unprecedented photocatalyst, Sm_2_EuSbO_7_, was successfully fabricated in this paper, through a high-temperature solid-state calcination method, which represented its first ever synthesis. Additionally, using the solvothermal method, the Sm_2_EuSbO_7_/ZnBiSbO_5_ heterojunction photocatalyst (SZHP) was fabricated, marking its debut in this study. XRD analysis confirmed that both Sm_2_EuSbO_7_ and ZnBiSbO_5_ exhibited pyrochlore-type crystal structures with a cubic lattice, belonging to the Fd3m space group. The crystal cell parameter was determined to be 10.5682 Å or 10.2943 Å for Sm_2_EuSbO_7_ or ZnBiSbO_5_, respectively. The band gap width measured for Sm_2_EuSbO_7_ or ZnBiSbO_5_ was 2.73 eV or 2.61 eV, respectively. Under visible light irradiation for 150 min (VLTI-150 min), SZHP exhibited remarkable photocatalytic activity, achieving 100% removal of parathion methyl (PM) concentration and 99.45% removal of total organic carbon (TOC) concentration. The kinetic constant (*k*) for PM degradation and visible light illumination treatment was determined to be 0.0206 min^−1^, with a similar constant *k* of 0.0202 min^−1^ observed for TOC degradation. Remarkably, SZHP exhibited superior PM removal rates compared with Sm_2_EuSbO_7_, ZnBiSbO_5_, or N-doped TiO_2_ photocatalyst, accompanied by removal rates 1.09 times, 1.20 times, or 2.38 times higher, respectively. Furthermore, the study investigated the oxidizing capability of free radicals through the use of trapping agents. The results showed that hydroxyl radicals had the strongest oxidative capability, followed by superoxide anions and holes. These findings provide a solid scientific foundation for future research and development of efficient heterojunction compound catalysts.

## 1. Introduction

To meet the incremental international demand for foodstuffs, more and more pesticides are used to boost crop yields in most developing countries [1,2,3,4]. It is worth mentioning that organophosphate pesticides (OEPs) are extensively utilized for their cost effectiveness and efficacy in managing pests, weeds, and diseases [5,6,7]. However, it is worth noting that fewer than 1% of the applied OEPs in agricultural production effectively fulfill their intended function of pest control. Consequently, a significant portion of the remaining OEPs remains in the soil, leading to the pollution of water resources [8,9]. Addressing the pressing need to efficiently eliminate pesticide residues from sewage and ensure the purification of water resources is of utmost importance.

Parathion methyl (PM), one of the most widely used OEPS, has been studied for its removal from water using various methods. Nevertheless, each of the methods has its limitations. Chemical oxidation, despite its effectiveness, comes with high costs and the risk of introducing additional toxic pollutants into water [10]. Meanwhile, biodegradation techniques are limited to treating diluted wastewater [11,12,13]. Similarly, methods like adsorption, precipitation, and membrane filtration present challenges in terms of operational costs, implementation complexities, and overall efficacy [14,15,16,17].

To address these limitations, photocatalytic technology has emerged as a potential approach for effluent treatment [18,19,20,21,22]. Photocatalytic technology utilizes solar energy to drive photocatalysts and realize the decomposition of water to produce free radicals with highly oxidative and reductive properties [23,24]. The free radicals can effectively remove organic pollutants without generating secondary pollution. Additionally, the photocatalyst has the advantage of being recyclable as well [25]. Therefore, the key to achieving commercial industrialization lies in the development of optimal photocatalysts that exhibit superior efficiency, significant specific surface area, maximum utilization of solar energy, and recyclability.

Owing to the wide band gaps, conventional metal oxide photocatalytic materials, namely titanium dioxide and zinc oxide, are mainly restricted to utilizing ultraviolet radiation, which dominates merely 5% of the solar energy wavelength range [26,27,28,29,30,31]. Thus, the remaining optical energy is wasted. To realize the utilization of the rest of the energy (43%), it is feasible to construct composite materials [32,33]. The composite materials have shown significant improvements in sunlight absorption range and photocatalytic performance (PCP) compared with single metal oxide catalysts [34,35,36]. In accordance with the conclusions drawn from previous investigations, AB_2_O_5_ and A_2_B_2_O_7_ mixtures have demonstrated superior PCP under visible light irradiation (VLTI) [37,38,39,40,41]. Karimi et al. have executed a study about the photocatalytic degradation (PDD) of metronidazole using Fe_2_TiO_5_ under UV-Vis lamp irradiation. Similarly, Zhang et al. made a indagation of the impressive PDD potential of La_2_Ce_2_O_7_ powders in the presence of methyl orange [42,43].

In previous work, we investigated the potential of structural modification on Bi_2_InTaO_7_, a visible light-responsive photocatalyst with a stable pyrochlore structure [44]. Additionally, we have drawn inspiration from previous studies on ZnO to guide our research. For example, Selvaraj et al. conducted a synthesis of Sm-doped ZnO and observed a prominent betterment in the PDD of methyl blue compared with pure ZnO. This finding suggests that the incorporation of Sm into ZnO has the potential to enhance its PCP [45]. Likewise, Zong et al. synthesized Eu-doped ZnO and observed a notable enhancement in the PDD of methyl orange in relation to pristine ZnO. This signifies that the incorporation of Eu can considerably ameliorate the PDD of organic dyes [46]. Furthermore, Nasser et al. synthesized Sb-doped ZnO, which demonstrated remarkable enhancements in the degradation of rhodamine B compared with undoped ZnO [47]. These studies collectively accentuate the effectiveness of rare earth element (such as Sm and Eu) and Sb introduction in enhancing the PCP of ZnO. Therefore, by replacing certain elements in Bi_2_InTaO_7_, such as Bi^3+^ with Sm^3+^, In^3+^ with Eu^3+^, and Ta^5+^ with Sb^5+^, we hypothesized that the novel Sm_2_EuSbO_7_ photocatalyst possessed improved PCP.

Additionally, the construction of heterostructure photocatalysts has shown great promise in enhancing PCP [48,49,50,51]. The interlaced band structure within the heterostructure forms a tight interface, establishing a strong internal electric field near the interface, thereby achieving efficient transfer and separation of light-activated carriers [52,53,54,55]. Zhao et al. developed a highly effective Ag_3_VO_4_/BiVO_4_ heterojunction photocatalyst, demonstrating superior effectiveness in degrading Bisphenol S as compared with pure Ag_3_VO_4_ and BiVO_4_ [56]. Lu et al. demonstrated that a Bi_2_O_3_/Bi_2_SiO_5_ heterojunction photocatalyst achieved a high photodegradation rate for organic pollutants [57].

Based on these insights, we designed and prepared a novel heterostructure photocatalytic material, Sm_2_EuSbO_7_/ZnBiSbO_5_, with the aim of significantly removing PM from pesticide wastewater under VLTI. The experimental findings demonstrated that the Sm_2_EuSbO_7_/ZnBiSbO_5_ heterojunction photocatalyst (SZHP) exhibited remarkable enhancement in the PDD of PM, achieving a removal rate of 91% within 100 min of VLTI. This highlights its superior PCP relative to NiO-Bi_2_MoO_6_ (a nearly 80% removal rate) and Ag-TiO_2_ nanoparticulate film (a nearly 60% removal rate) in parallel experimental scenarios [58,59].

In this research endeavor, we characterized the structures of pure-phase Sm_2_EuSbO_7_ and single-phase ZnBiSbO_5_ by means of an X-ray diffractometer (XRD), ultraviolet-visible (UV-Vis) spectrophotometer, Fourier transform infrared (FTIR) spectrometer, Raman spectrometer, X-ray photoelectron spectroscopy (XPS), transmission electron microscopy (TEM), and energy dispersive X-ray spectroscopy (EDS). Furthermore, we evaluated the performance of these photocatalysts, including SZHP, Sm_2_EuSbO_7_, ZnBiSbO_5_, and nitrogen-doped titanium dioxide (N-TO), by studying their degradation efficiency of PM in pesticide wastewater under VLTI. The main innovation of this study lies in the first synthesis of an innovative visible-light-responsive Sm_2_EuSbO_7_ photocatalyst with high PCP using the high-temperature solid-state calcination method. Furthermore, the findings of this study contribute significantly to the betterment of a highly effective and safe photocatalytic system for the PDD of PM in pesticide wastewater.

## 2. Results and Discussion

### 2.1. X-ray Diffraction Analysis

**Figure 1:**
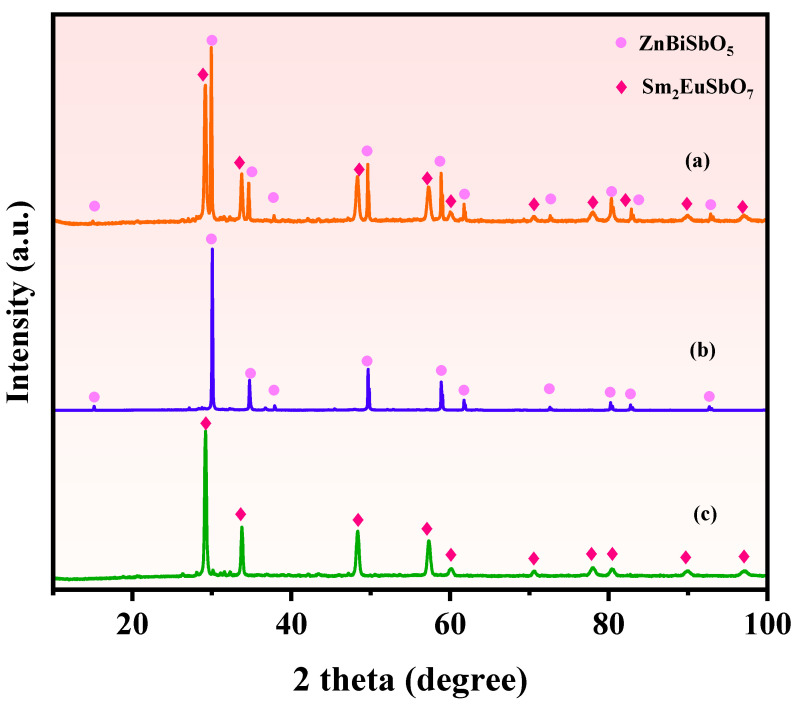
XRD spectra of as-fabricated specimen: (**a**) SZHP, (**b**) ZnBiSbO_5_, (**c**) Sm_2_EuSbO_7_.

**Figure 2:**
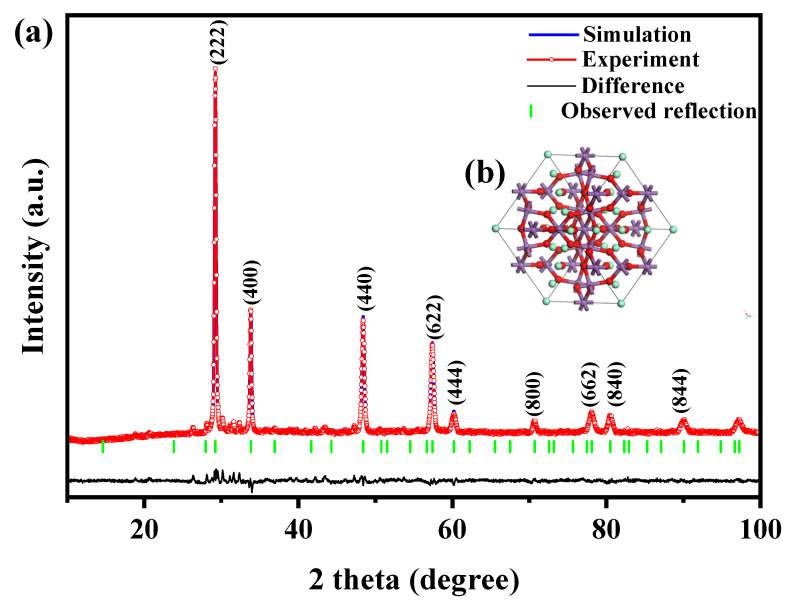
(**a**) XRD patterns and Rietveld refinement and (**b**) The atomic structure (Red atom: O, purple atom: Eu or Sb, green atom: Sm) of Sm_2_EuSbO_7_.

**Figure 3:**
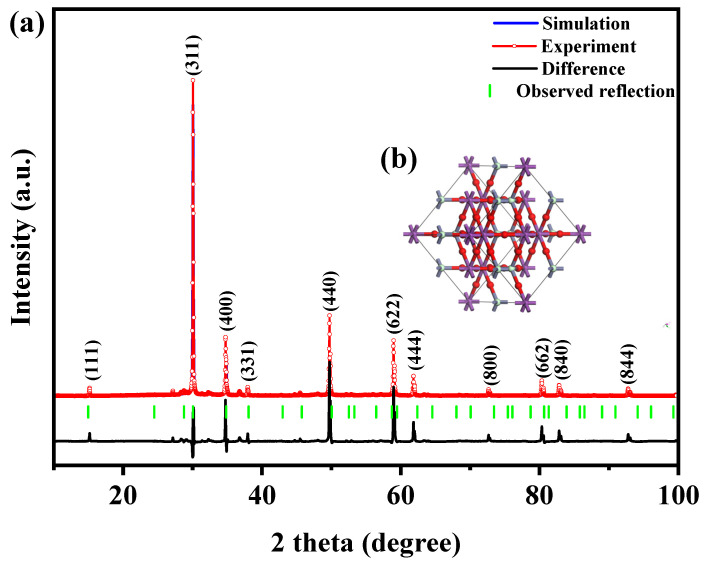
(**a**) XRD patterns and Rietveld refinement and (**b**) the atomic structure (Red atom: O, green atom: Zn, purple atom: Bi or Sb) of ZnBiSbO_5_.

Figure 1a showcases the XRD pattern of the Sm_2_EuSbO_7_/ZnBiSbO_5_ heterojunction photocatalyst (SZHP), which was fabricated using the solvothermal method. In Figure 1b,c, the XRD patterns of ZnBiSbO_5_ and Sm_2_EuSbO_7_ prepared via the solid-state calcination method are presented. The prominent peaks observed in the XRD pattern of SZHP coincide with those of both ZnBiSbO_5_ and Sm_2_EuSbO_7_, indicating the successful synthesis of SZHP. The experiment data of Sm_2_EuSbO_7_ and ZnBiSbO_5_ compounds were subjected to Rietveld refinement using the Materials Studio program. The refinement results for Sm_2_EuSbO_7_ and ZnBiSbO_5_ compounds are presented in Figure 2a and Figure 3a correspondingly. The Rietveld refinement of Sm_2_EuSbO_7_ (R_P_ = 2.42%) and ZnBiSbO_5_ (R_p_ = 15.81%) confirmed excellent agreement in the comparison of experimental and calculated intensities, indicating a high level of precision in characterizing the pyrochlore-type structure. Meanwhile, the results demonstrated that both compounds were single-phase and cubic lattice with the space group Fd3m. According to the interplanar crystal spacing *d* of every diffraction peak, the diffraction angle of every diffraction peak, and the wavelength of the copper target (1.5406 Å) in the X-ray diffractometer, the crystal lattice dimensions of Sm_2_EuSbO_7_ and ZnBiSbO_5_ were determined to be 10.5862 Å and 10.2943 Å, respectively. The refinement model included O atoms, which showed excellent agreement in the comparison of experimental and calculated intensities. Figure 2b and Figure 3b illustrate the atomic structures of Sm_2_EuSbO_7_ and ZnBiSbO_5_ severally. The atoms’ positions and the geometric factors of Sm_2_EuSbO_7_ and ZnBiSbO_5_ are provided in Table 1 and Table 2, respectively. These observations support the notion that the synthesized compounds possess excellent structural stability and could potentially be applied as efficient photocatalysts in various fields [60].

Pyrochlore-type A_2_B_2_O_7_ compounds are renowned for their exceptional structural stability. The x co-ordinates of the O(1) atom carries significant weight in the analysis of the crystal architecture and is universally acknowledged as a fundamental parameter for characterizing the structural properties, indicating its significance in characterizing the material. The x value of 0.375 signifies the equivalence between the six A-O(1) connection dimensions and the two A-O(2) connection dimensions [61]. It is observed that deviations from x = 0.375 indicate a distortion in the MO_6_ (M = Eu^3+^ and Sb^5+^) octahedra, implying a crystal structure distortion in Sm_2_EuSbO_7_ [61]. Carrier separation realizes a prominent role in the efficient PDD of PM under VLTI, as it prevents the recombination of light-activated carriers. In some photocatalytic materials, including BaTi_4_O_9_ and Sr_2_M_2_O_7_ (M = Nb^5+^ and Ta^5+^), a local distortion of MO_6_ octahedra has been reported to prevent charge recombination and improve PCP [62,63]. Therefore, the distortion of MO_6_ (M = Eu^3+^ and Sb^5+^) octahedra in Sm_2_EuSbO_7_ could potentially enhance PCP. Sm_2_EuSbO_7_ has a 3D interconnected framework of common-corner MO_6_ (M = Eu^3+^ and Sb^5+^) octahedra, which are connected into chains by Sm^3+^ ions. The crystal structure of Sm_2_EuSbO_7_ displays two types of Sm-O connection dimensions, where the six Sm-O(1) connection dimensions (2.491 Å) are longer than the two Sm-O(2) connection dimensions (2.230 Å). The six M-O(1) (M = Eu^3+^ and Sb^5+^) connection dimensions were 2.020 Å, and the M-Sm (M = Eu^3+^ and Sb^5+^) connection dimension was 3.642 Å. The M-O-M (M = Eu^3+^ and Sb^5+^) interatomic angle was 128.640^◦^, while the Sm-M-Sm (M = Eu^3+^ and Sb^5+^) interatomic angle was 135.00^◦^ in the crystal architecture of Sm_2_EuSbO_7_. The Sm-M-O (M = Eu^3+^ and Sb^5+^) interatomic angle was 139.203^◦^. The M-O-M interatomic angle affects the delocalization of the excitation state. A smaller deviation from 180° in the M-O-M angle enhances the luminescent function. The M-O-M interatomic angle in Sm_2_EuSbO_7_ affects PCP by influencing the migration of light-activated electron–hole pairs, which affects their potential to reach surface-active centers of photocatalytic substances. Additionally, the larger Sm-Eu-O interatomic angle or Sm-Sb-O interatomic angle in Sm_2_EuSbO_7_ increases its PCP. The effect of PM degradation under VLTI with Sm_2_EuSbO_7_ as a photocatalyst mainly depends on its crystalline and electronic structure.

### 2.2. FTIR Analysis

**Figure 4:**
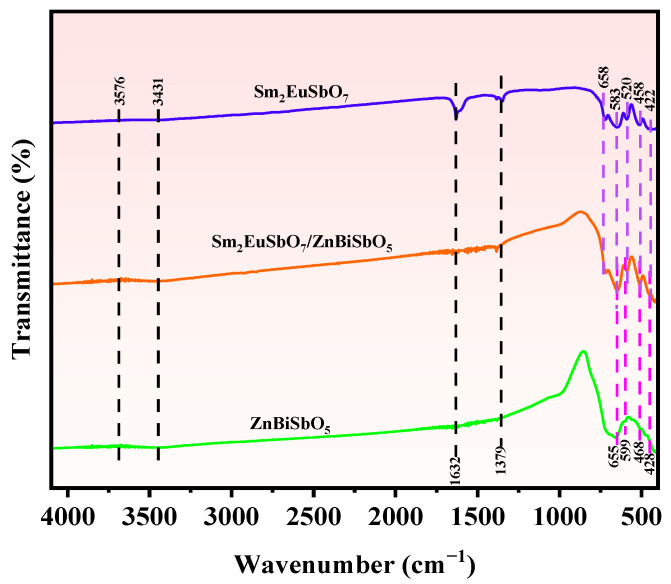
FTIR spectra of Sm_2_EuSbO_7_, ZnBiSbO_5_, and SZHP.

To determine the presence of functional groups and chemical bonding in SZHP, as well as Sm_2_EuSbO_7_ and ZnBiSbO_5_, an FTIR spectrum was obtained using an FTIR spectrometer. Figure 4 illustrates the FTIR spectrum of SZHP, prepared via the solvothermal method, as well as Sm_2_EuSbO_7_ and ZnBiSbO_5_ prepared through the solid-state calcination method. The FTIR spectrum exhibits several characteristic absorption peaks. These include peaks associated with Sm-O, Zn-O, Bi-O, Sb-O, Sb-O-Sb, and Eu-O bonds. The stretching oscillation of Sm-O is identified at 520 cm^−1^ [64,65,66], while the bending oscillation of Zn-O occurs at 468 cm^−1^ [67]. The stretching oscillation of Bi-O is associated with peaks at 428 cm^−1^ [68,69], and the bending oscillation of Sb-O is manifest at 458 cm^−1^, 583 cm^−1^, and 599 cm^−1^ [70]. The bending oscillation of Sb-O-Sb is represented by peaks at 655 cm^−1^ and 658 cm^−1^ [71]. Additionally, the stretching oscillation of Eu-O is detected at 422 cm^−1^ [72,73]. Furthermore, the broad peak observed at 3431 to 3576 cm^−1^ indicates the stretching oscillations of O-H groups from chemisorbed water molecules [74,75]. The peak at 1632 cm^−1^ is in agreement with the bending oscillation manner of these O-H groups [76]. The multiple bands observed at 1379 to 1632 cm^−1^ can be in association with the oscillations of C-H bonds from adsorbed water [77,78].

### 2.3. Raman Analysis

**Figure 5:**
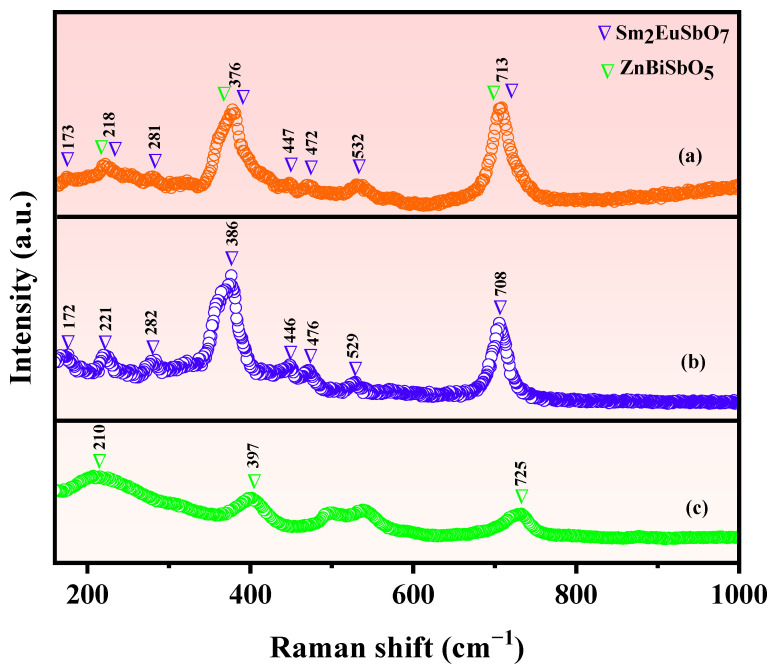
Raman spectra of (**a**) SZHP, (**b**) Sm_2_EuSbO_7_, and (**c**) ZnBiSbO_5_.

In order to obtain the interactions among the various chemical bonds within SZHP, Sm_2_EuSbO_7_, and ZnBiSbO_5_, a Raman spectrum was obtained with a Raman spectrometer. Figure 5 depicts the Raman spectrum of SZHP prepared by the solvothermal method, as well as Sm_2_EuSbO_7_ and ZnBiSbO_5_ prepared via the solid-state calcination method. The Raman spectrum of Sm_2_EuSbO_7_ exhibits characteristic modes, including the A_g_ internal Sm-O stretching modes at 172 cm^−1^ [79], as well as the E_1g_ main vibrational modes at 375 cm^−1^, 446 cm^−1^, and 703 cm^−1^, in association with the stretching oscillation of Eu-O bonds [80]. Additionally, peaks at 221 cm^−1^, 282 cm^−1^, 476 cm^−1^, and 529 cm^−1^ can be designated to the bend oscillation of Sb-O and Sb-O-Sb [81,82]. The Raman spectra of ZnBiSbO_5_ reveal a broad band at 210 cm^−1^, 390 cm^−1^, and 730 cm^−1^, which can be in association with the stretching oscillations of Bi-O, Sb-O, and Zn-O, respectively [81,82,83,84,85,86]. Notably, the Raman spectra of SZHP exhibited strong peaks encompassing the distinct absorption peaks of both Sm_2_EuSbO_7_ and ZnBiSbO_5_, including peaks at 173 cm^−1^, 218 cm^−1^, 281 cm^−1^, 377 cm^−1^, 442 cm^−1^, 472 cm^−1^, 532 cm^−1^, and 707 cm^−1^.

### 2.4. UV-Vis Diffuse Reflectance Spectra

**Figure 6:**
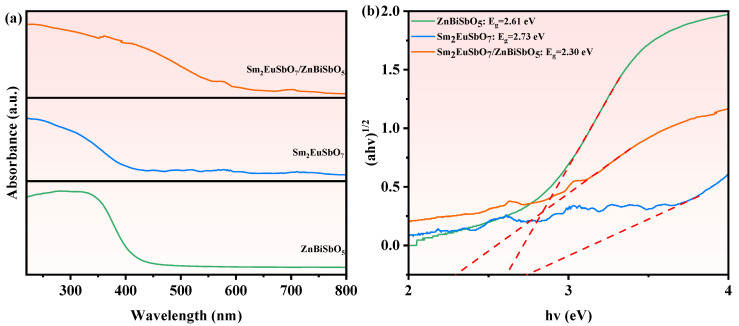
(**a**) The UV-Vis diffuse reflectance spectra and (**b**) correlative diagram of (*αhν*) ^1/2^ and *hν* of the synthesized SZHP, Sm_2_EuSbO_7_, and ZnBiSbO_5_.

To scrutinize the band architecture of the synthesized materials, an absorption spectrum was acquired. Figure 6a illustrates the absorption spectrum of Sm_2_EuSbO_7_ and ZnBiSbO_5_, both synthesized through solid-phase sintering, along with SZHP, which was prepared using the solvothermal method. The absorption boundary of new photocatalytic materials Sm_2_EuSbO_7_ and ZnBiSbO_5_ was measured to be at 434 nm and 455 nm severally. The absorption boundary of SZHP was 523 nm, displaying prominent red-shift relative to ZnBiSbO_5_ and Sm_2_EuSbO_7_. It was demonstrated that SZHP showcased superior light-absorbing properties relative to ZnBiSbO_5_ and Sm_2_EuSbO_7_.

The energy band gaps of the light-activated catalysts can be evaluated by identifying the dot of intersection between the photonic energy hν axis and the line extrapolated from the rectilinear portion of the absorption boundary, which is represented by the Kubelka–Munk function (1). This function is utilized for this purpose [87,88].

(1) $$\frac{{\left[1-R_{d}(h\nu )\right]}^{2}}{2R_{d}(h\nu )}=\frac{\alpha (h\nu )}{S}$$

Here, S represents the scattering determinant, R_d_ refers to the diffuse reflectivity, and α denotes the light absorption determinant of simulation radiation.

The photon ingestion near the energy band boundary of the light-activated catalysts followed Equation (2) [89,90]:

(2) $$\alpha h\nu =A{\left(h\nu -E_{g}\right)}^{n}$$

In this mathematical model, A, α, $E_{g}$, and *ν* correspond to the merical number of proportionality, optical ingestion parameter, energy gap, and photonic frequency, respectively. The merical number of n controls the optical transition behavior of the photocatalyst. To determine E_g_ and *n*, the following steps were taken: (1) depict ln(αhν) versus $ln(h\nu -E_{g})$ establishing a rough approximation of $E_{g}$; (2) deduce the merical number of n guided by the slope of the diagram; (3) optimize the merical number of E_g_ by depicting ${\left(\alpha h\nu \right)}^{1/n}$ versus hν and extrapolating the mapping to ${\left(\alpha h\nu \right)}^{1/n}$ = 0. An estimation of the valence-conduction gap, $E_{g}$, for Sm_2_EuSbO_7_ or ZnBiSbO_5_ was obtained using the straightforward approach (1240/transition wavelength λ).

The E_g_ merical number of Sm_2_EuSbO_7_ or ZnBiSbO_5_ was evaluated to be 2.85 eV or 2.72 eV severally. The precise band gap width of Sm_2_EuSbO_7_ or ZnBiSbO_5_ was measured using the indirect method (Equation (2)) with the estimated E_g_ merical number. According to Figure 6b, the computed E_g_ merical numbers for Sm_2_EuSbO_7_ and ZnBiSbO_5_ were 2.73 eV and 2.61 eV severally. The determined merical number of n was about 2 for Sm_2_EuSbO_7_ and ZnBiSbO_5_, indicating that the light-induced transition was indirectly allowed. Similarly, based on Figure 6b, the E_g_ value for SZHP was 2.30 eV and the optical transition was indirectly allowed [91].

The E_g_ value of SZHP was smaller compared with that of Sm_2_EuSbO_7_ and ZnBiSbO_5_, which can be credited to the inclusion of interface effects and electron transport effects during the formation of the heterojunction. In SZHP, when Sm_2_EuSbO_7_ and ZnBiSbO_5_ came into contact and formed an interface, there were intrinsic differences in the properties of these two photocatalytic materials [92]. These differences resulted in band bending and energy level shifts at the interface, known as interface effects. As a consequence, the band structure was modified, resulting in a decrease in the band gap width. Besides the interface effects, electron charge migration behavior came to pass at the interface of SZHP due to the contrasting electron affinities and ionization potentials of its constituent components [93]. This charge transfer caused energy level shifts in the corresponding bands, further contributing to the band narrowing [94]. Therefore, SZHP demonstrated a narrower band gap compared with the separate Sm_2_EuSbO_7_ and ZnBiSbO_5_ components within the heterojunction. This band-narrowing effect was advantageous as it expanded the absorption range of the heterojunction photocatalytic materials, enhanced its photocatalytic activity, and promoted the enhanced dissociation and conveyance of optically generated electron–hole pairs, consequently increasing the catalytic reaction efficiency. By incorporating these interface effects and electron transfer effects, SZHP exhibited superior performance as a photocatalyst compared with Sm_2_EuSbO_7_ and ZnBiSbO_5_ in their individual forms. These findings support the potential application of SZHP as an efficient photocatalyst in various catalytic processes.

### 2.5. Property Characterization of Sm_2_EuSbO_7_/ZnBiSbO_5_ Heterojunction Photocatalyst

**Figure 7:**
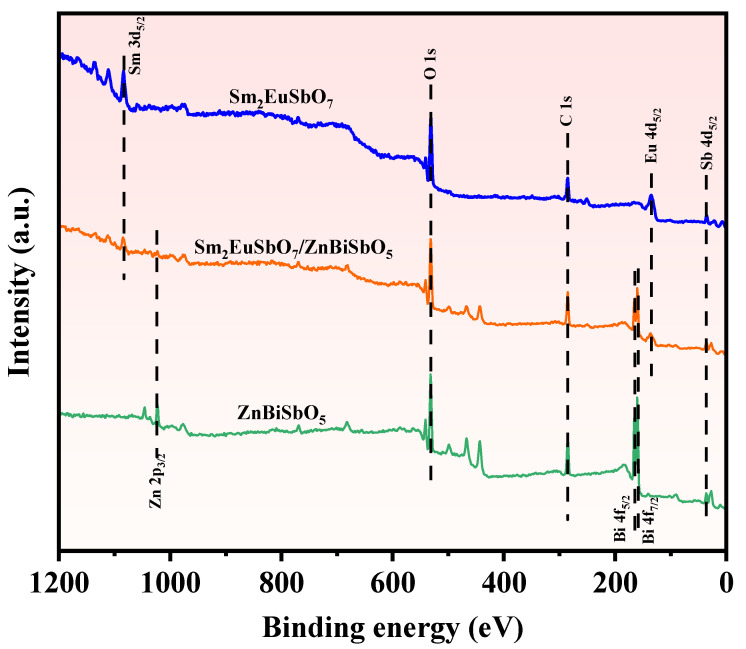
The XPS full spectrum of the synthesized SZHP, Sm_2_EuSbO_7_, and ZnBiSbO_5_.

To investigate the element constitution and oxidation states of SZHP, prepared via the solvothermal method as well as Sm_2_EuSbO_7_, and ZnBiSbO_5_, prepared through the solid-state calcination method, XPS was employed. The integrated spectrum in Figure 7 clearly demonstrates the presence of Sm, Eu, Sb, Zn, Bi, and O elements in SZHP. A carbon peak was observed and attributed to adventitious hydrocarbon, which served as a calibration reference. Upon comparing the spectrum of Sm_2_EuSbO_7_ to that of SZHP, distinctive zinc and bismuth signals were observed in the latter, highlighting the introduction of ZnBiSbO_5_ in SZHP.

**Figure 8:**
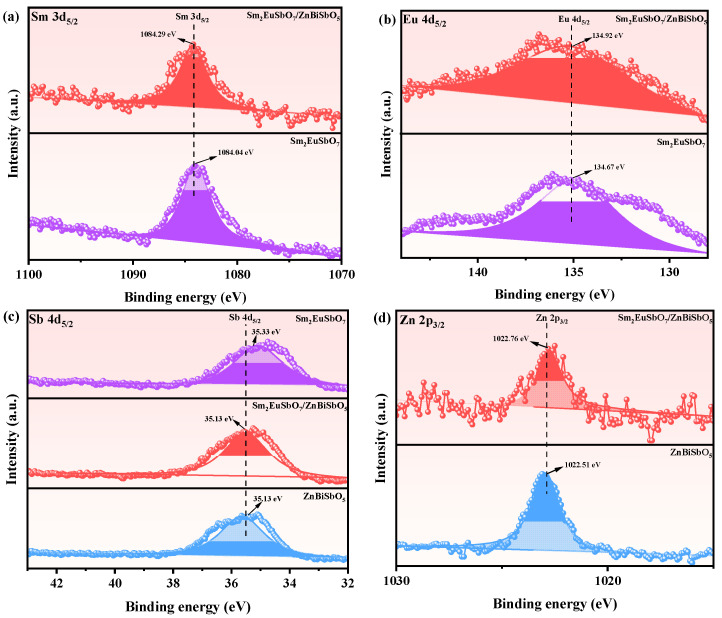

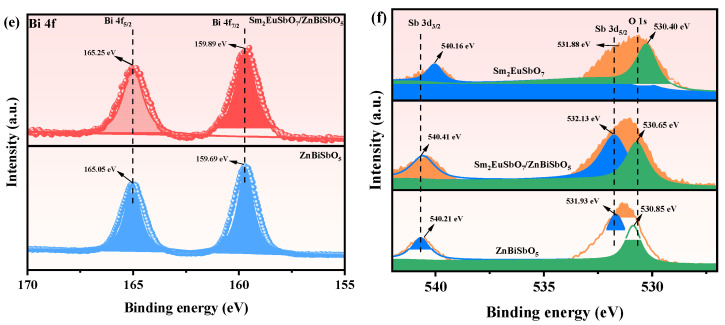
The corresponding XPS spectra of (**a**) Sm 3d, (**b**) Eu 4d, (**c**) Sb 4d, (**d**) Zn 2p, (**e**) Bi 4f, and (**f**) O 1s of SZHP, Sm_2_EuSbO_7_, and ZnBiSbO_5_.

Figure 8a–e presents the deconvoluted spectral peaks of Sm 3d, Eu 4d, Sb 4d, Zn 2p, and Bi 4f in Sm_2_EuSbO_7_ and ZnBiSbO_5_. These peaks were observed at 1084.04 eV (Sm 3d_5/2_), 134.67 eV (Eu 4d_5/2_), 35.08 and 35.13 eV (Sb 4d_5/2_), 1022.56 eV (Zn 2p_3/2_) and 159.69 (Bi 4f_7/2_) and 165.05 eV (Bi 4f_5/2_). Intriguingly, these peaks exhibit slight shifts towards higher binding energies, such as 1084.29 eV (Sm 3d_5/2_), 134.92 eV (Eu 4d_5/2_), 35.33 eV (Sb 4d_5/2_), 1022.76 eV (Zn 2p_3/2_), 159.89 eV (Bi 4f_7/2_) and 165.29 eV (Bi 4f_5/2_) in SZHP [95,96]. These observed shifts provide evidence for a robust interfacial interaction between Sm_2_EuSbO_7_ and ZnBiSbO_5_, suggesting a possible electron transfer and delocalization phenomenon within the heterojunction photocatalytic materials. The presence of such interfacial interaction strengthens the overall efficiency and performance of the materials in photochemical processes. Moreover, the spin-orbit separation values between Bi 4f_7/2_ and Bi 4f_5/2_ were determined to be 5.36 eV for both ZnBiSbO_5_ and SZHP, indicating the exclusive presence of Bi^3+^ [97].

Figure 8f illustrates the deconvoluted O 1s spectrum of SZHP, Sm_2_EuSbO_7_, and ZnBiSbO_5_. The O 1s orbital coordinates with the peaks at 530.40 eV, 530.65 eV, and 530.85 eV. The distinct shifts further confirm the interfacial interaction between Sm_2_EuSbO_7_ and ZnBiSbO_5_. Moreover, the recorded peaks at 531.88 eV and 532.13 eV, as well as 531.93 eV, along with 540.16 eV, 540.41 eV, and 540.21 eV, can be put down to the Sb 3d_5/2_ and Sb 3d_3/2_ orbitals, respectively. These findings indicate the presence of specific electronic transitions and provide valuable insights into the electronic structure of the materials under investigation. Likewise, the spin-orbit separation value between Sb 3d_5/2_ and Sb 3d_3/2_ was observed to be 8.28 eV for ZnBiSbO_5_, Sm_2_EuSbO_7_, and SZHP, confirming the exclusive presence of Sb^5+^ [98,99].

In accordance with the outcomes obtained from the XPS analysis, it can be inferred that the oxidation states of Sm, Eu, Sb, Zn, Bi, and O ions within the material are +3, +3, +5, +2, +3, and −2 severally. This empirical evidence proffers valuable insights into the valence states of the constituent elements and their contributions to the overall chemical composition. Further analysis of the surface composition indicated that the average atomic ratio of Sm:Eu:Sb:Zn:Bi:O was determined to be 785:391:773:414:402:7235. The atomic ratio of Sm:Eu:Sb or Zn:Bi:Sb in SZHP was 2.03:1.01:1.00 and 1.07:1.04:1.00 severally. Clear evidence of the absence of any other phase was observed as no shoulder or broadening in the XPS spectrum of both Sm_2_EuSbO_7_ and ZnBiSbO_5_ samples. This finding indicates that the materials were in pure- and single-phase states.

**Figure 9:**
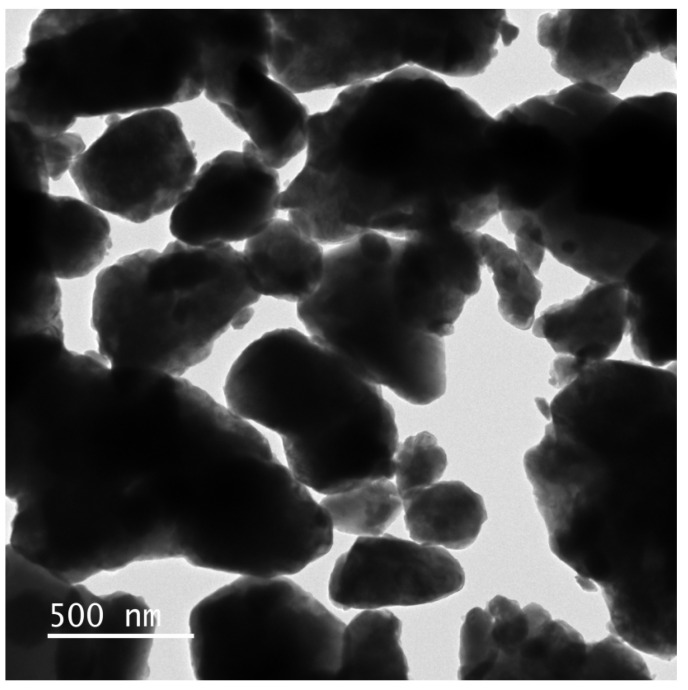
TEM image of SZHP.

**Figure 10:**
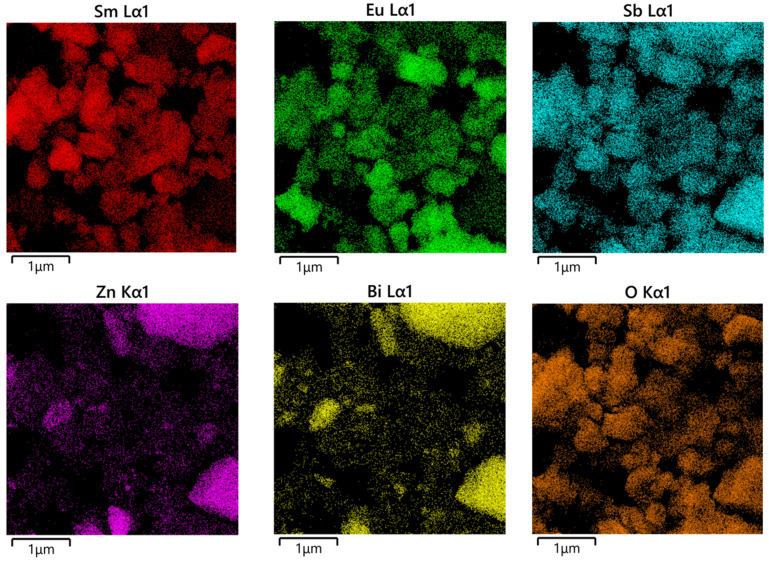
EDS element mapping of SZHP (Sm, Eu, Sb, and O from Sm_2_EuSbO_7_ and Zn, Bi, Sb, and O from ZnBiSbO_5_).

**Figure 11:**
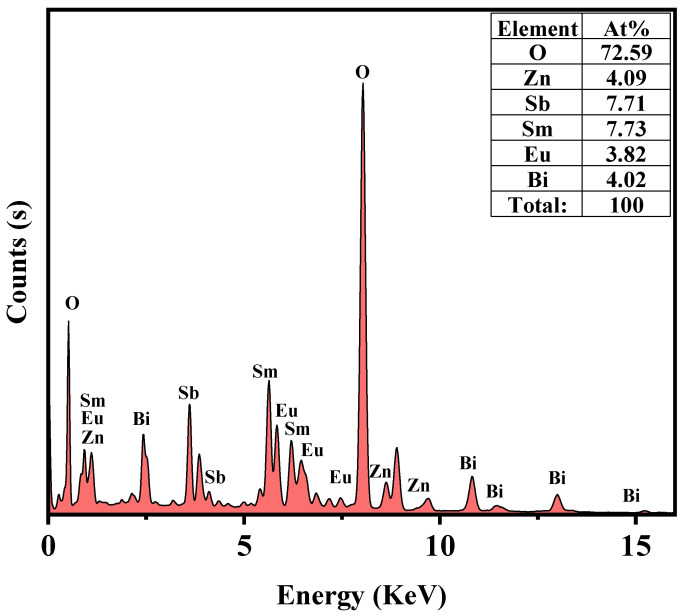
The EDS spectrum of SZHP.

The SZHP sample, which was prepared using the solvothermal method, was inspected through transmission electron microscopy (TEM) and energy-dispersive X-ray spectroscopy (EDS). Figure 9 and Figure 10 illustrate the TEM scheme and EDS element mapping of SZHP (Sm, Eu, Sb, and O from Sm_2_EuSbO_7_ and Zn, Bi, Sb, and O from ZnBiSbO_5_) severally. The EDS spectrum of SZHP is presented in Figure 11. Upon observation of Figure 9 and Figure 10, it is evident that the larger microboulders correspond to ZnBiSbO_5_, while the smaller globular microboulders belong to Sm_2_EuSbO_7_. The TEM images further reveal that the microboulders of ZnBiSbO_5_ are enveloped by the microboulders of Sm_2_EuSbO_7_, indicating successful synthesis of SZHP. The particle dimension of Sm_2_EuSbO_7_ was assessed to be approximately 380 nm, whereas the larger microboulder dimension of ZnBiSbO_5_ measured around 1300 nm [100].

The EDS element mapping of SZHP in Figure 10 confirms the presence of samarium, europium, antimony, zinc, bismuth, and oxygen elements within SZHP. This observation indirectly supports the coexistence of both Sm_2_EuSbO_7_ and ZnBiSbO_5_ in SZHP. These findings conform to the XPS data of SZHP depicted in Figure 7 and Figure 8. The EDS spectral distribution, which is manifested in Figure 11, revealed that the atomic ratio of Sm:Eu:Sb:Zn:Bi:O in SZHP was determined to be 773:382:771:409:402:7259. This finding matches the results obtained from XPS analysis, reinforcing the reliability of the obtained data. The atomic ratio of Sm_2_EuSbO_7_:ZnBiSbO_5_ was close to 1.2:1, which was consistent with the approximate molar ratio of the two compounds used in the experiment. Based on these findings, one can conclude that SZHP was successfully synthesized with a high level of purity under the specific preparation conditions employed.

### 2.6. Property Activity

**Figure 12:**
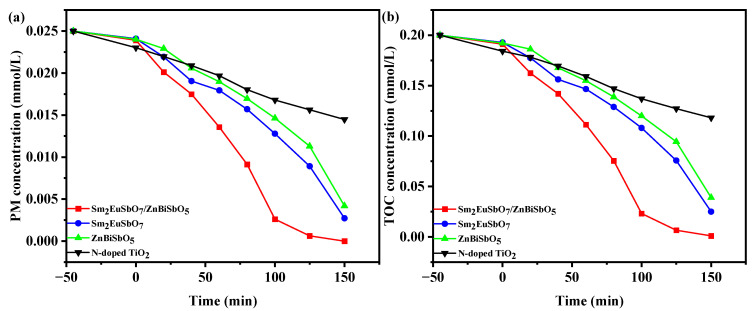
Saturation variation profiles of (**a**) PM and (**b**) TOC during the photodegradation of PM with SZHP, Sm_2_EuSbO_7_, ZnBiSbO_5_, or N-TO as the photocatalyst under VLTI.

The present study investigated the concentration variability profiles of PM during PDD under VLTI using various photocatalysts. The photocatalysts employed in this investigation consisted of SZHP, Sm_2_EuSbO_7_, ZnBiSbO_5_, and N-TO. The concentration patterns of PM in pesticide wastewater over time under VLTI are depicted in Figure 12a. The obtained results clearly demonstrate a consistent decline in the concentration of PM corresponding to the extended period of irradiation.

Analysis of the data obtained from Figure 12a revealed that the use of SZHP as the photocatalyst resulted in complete removal of PM within pesticide wastewater, achieving a reaction velocity of 2.78 × 10^−9^ mol·L^−1^·s^−1^ and a photonic efficiency (PNE) of 0.0580% after 150 min of VLTI. The other photocatalysts were subjected to similar experimental conditions. When Sm_2_EuSbO_7_ was utilized as the photocatalyst, it achieved a remarkable PM removal rate of 89.1%, demonstrating a reaction velocity of 2.48 × 10^−9^ mol·L^−1^·s^−1^ and PNE of 0.0521%. Likewise, in the case of ZnBiSbO_5_ as the photocatalyst, a significant PM removal rate of 83.2% was achieved in pesticide wastewater, accompanied by a reaction velocity of 2.31 × 10^−9^ mol·L^−1^·s^−1^ and PNE of 0.0485%. Lastly, N-TO exhibited a PM removal rate of 42%, indicating a reaction velocity of 1.17 × 10^−9^ mol·L^−1^·s^−1^ and PNE of 0.0246%.

Based on the above findings, it can be concluded that SZHP demonstrated the highest photodegradation efficiency for PM compared with the other photocatalysts. Additionally, the photodegradation efficiency achieved using Sm_2_EuSbO_7_ as the photocatalyst was superior to that of ZnBiSbO_5_ and N-TO. Furthermore, the photodegradation efficiency obtained with ZnBiSbO_5_ as the photocatalyst surpassed that of N-TO. These findings indicate that SZHP exhibits enhanced photocatalytic activity under VLTI compared with individual Sm_2_EuSbO_7_, ZnBiSbO_5_, and N-TO materials.

Moreover, upon comparing the PM degradation velocity achieved through 150 min of VLTI, it becomes clear that the effectiveness of SZHP surpassed that of Sm_2_EuSbO_7_ by 1.11 times, ZnBiSbO_5_ by 1.20 times, and N-TO by 2.38 times.

The saturation variation profiles of TOC throughout the PDD of PM in pesticide effluent under VLTI using different photocatalysts are presented in Figure 12b. The investigated photocatalysts include SZHP, Sm_2_EuSbO_7_, ZnBiSbO_5_, and N-TO. As illustrated in Figure 12b, the TOC concentration exhibited reduction as the VLTI time increased.

The analysis of Figure 12b demonstrates that following a 150-min duration of VLTI, the degradation rates of TOC within pesticide wastewater were found to be 99.45%, 87.5%, 80.55%, and 40.95% when employing SZHP, Sm_2_EuSbO_7_, ZnBiSbO_5_, and N-TO as photocatalysts, respectively, for the treatment of PM.

The superior PDD efficiency and TOC removal efficiency of SZHP compared with Sm_2_EuSbO_7_, ZnBiSbO_5_, and N-TO could primarily be traced back to the efficient segregation of photoproduced electrons and photoproduced holes, and suppression reassociation of photoproduced carriers. The formation of the heterostructure photocatalytic materials facilitated the dimensional segregation of carriers within distinct regions of the photocatalytic materials, effectively mitigating their recombination [101,102,103]. The effective separation of photoproduced carriers and the suppression of recombination processes had a significant impact on enhancing the capability of PDD and TOC removal. Additionally, the formation of heterojunctions brought about alterations in the band structure, including the shift of band edge positions and modulation of band gaps. These modifications expanded the absorption range, allowing for more efficient excitation of electrons to the conduction band by absorbing a broader spectrum of photons [104,105,106]. Through these controlled band structure adjustments, SZHP exhibited higher light absorption and enhanced PCP. Moreover, the heterojunction interface provided additional active sites and surface defects, which played a crucial role in chemical reactions. These inherent features contributed to enhanced PDD efficiency and TOC removal. The interface effects offered opportunities for enhanced catalyst interactions, potentially resulting in synergistic effects and increased catalytic activity. In summary, the superior photodegradation efficiency and TOC removal efficiency of SZHP stemmed from its ability to effectively separate and suppress the recombination of light-activated electron–hole pairs, and also stemmed from the adjustments in the band structure and the availability of additional active sites at the heterojunction interface. These attributes highlighted the potential of SZHP as a promising material for high-performance photocatalysis, with broad applications in enhancing photocatalytic reaction efficiency and energy conversion.

**Figure 13:**
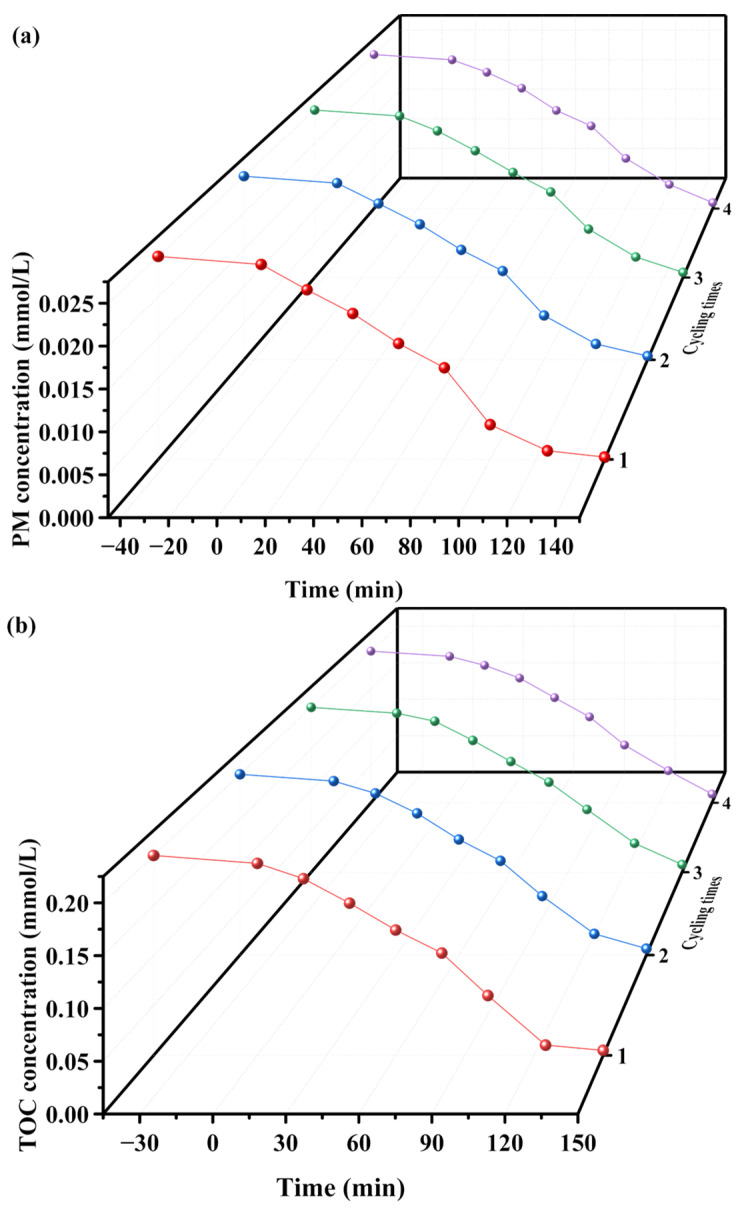
Concentration variation curves of (**a**) PM and (**b**) TOC during the photodegradation of PM in pesticide wastewater with SZHP as photocatalyst under VLTI for four cycle degradation tests (red line: first cycle, blue line: second cycle, green line: third cycle, purple line: fourth cycle).

The saturation variation profiles of PM and TOC during four consecutive degradation cycles using SZHP as a photocatalyst under VLTI were shown in Figure 13a,b, respectively [107]. After 150 min of VLTI, the removal rates of PM were found to be 98.94%, 97.96%, 96.88%, and 96.12% in the four cycles; the removal rates of TOC were found to be 97.5%, 96.45%, 95.3%, and 94.2% in the four cycles. These observations reveal the remarkable stability of SZHP during the degradation process. Additionally, a reduction of 2.82% in the PM degradation rate and a decrease of 3.3% in the TOC removal rate were observed across four consecutive degradation cycles. These findings suggest that SZHP exhibits remarkable structural stability and can be effectively reused as a photocatalyst.

To underscore the novelty and significance of our research in the realm of PM degradation through photocatalysis, a comprehensive comparative analysis was conducted. In Table 3, a comparative overview of pertinent studies in this field is presented, highlighting the superior PM degradation efficiency of SZHP. The results unequivocally demonstrate that when SZHP serves as the photocatalyst, it outperforms other catalysts in terms of PM degradation, emphasizing the notable catalytic potential of SZHP in this context. These findings underscore the pivotal role of SZHP in enhancing the rate of PM PDD, thereby contributing significantly to the field of photocatalysis.

**Figure 14:**
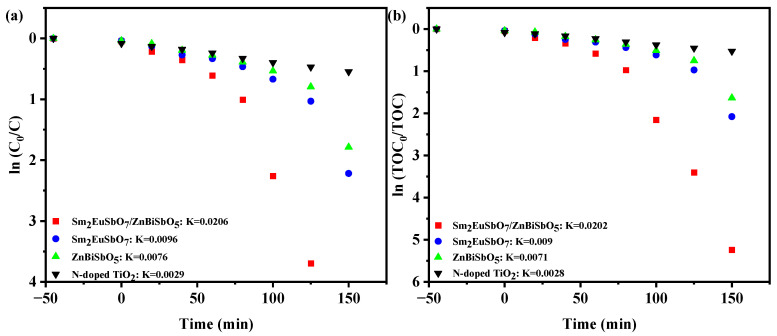
Identified first-order kinetic charts for (**a**) PM and (**b**) TOC during the photodegradation of PM with SZHP, Sm_2_EuSbO_7_, ZnBiSbO_5_, or N-TO as the photocatalyst under VLTI.

Figure 14 exhibits the first-order kinetic schematics of PM (Figure 14a) and TOC (Figure 14b) during the photodegradation of PM with various photocatalysts, including SZHP, Sm_2_EuSbO_7_, ZnBiSbO_5_, and N-TO, under VLTI. *K_C_* (the kinetic merical number derived through the dynamic curve of PM saturation and VLTI time) were 0.0206 min^−1^, 0.0096 min^−1^, 0.0076 min^−1^, and 0.0029 min^−1^ for SZHP, Sm_2_EuSbO_7_, ZnBiSbO_5_, and N-TO severally. Additionally, *K_TOC_* (the kinetic merical number derived through the dynamic curves of TOC saturation as a function of VLTI time) were determined to be 0.0202 min^−1^, 0.009 min^−1^, 0.0071 min^−1^, and 0.0028 min^−1^ for SZHP, Sm_2_EuSbO_7_, ZnBiSbO_5_, and N-TO severally. Notably, *K_TOC_* was lower than *K_C_* for all the catalysts, indicating the formation of intermediate species during the PDD process. SZHP displayed superior mineralization performance for PDD of PM as opposed to the other photocatalysts.

**Figure 15:**
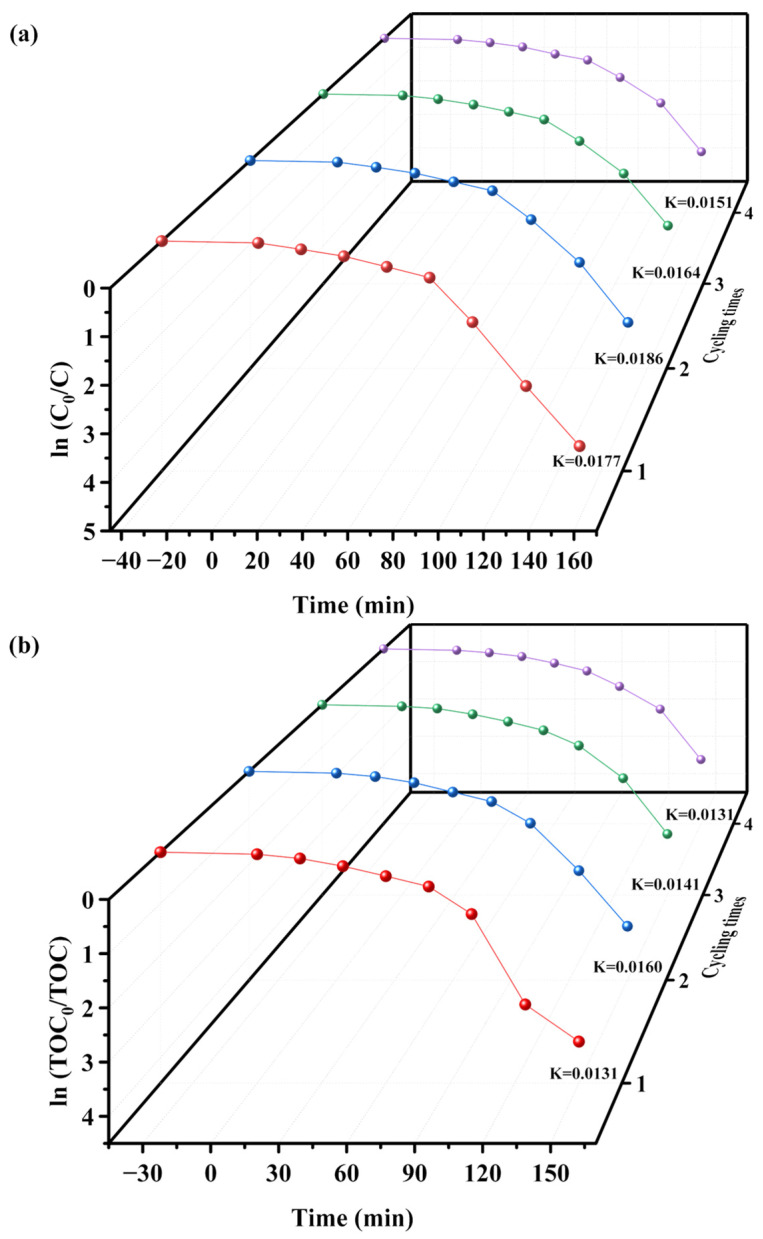
Identified first-order kinetic charts for (**a**) PM and (**b**) TOC during the photodegradation of PM with SZHP in the function of a photocatalytic material under VLTI for degradation evaluations spanning four cycles (red line: first cycle, blue line: second cycle, green line: third cycle, purple line: fourth cycle).

Figure 15 exhibits the first-order kinetic plots of PM (Figure 15a) and TOC (Figure 15b) during the photodegradation of PM with SZHP as the photocatalyst under VLTI in four consecutive degradation cycles. *K_C_* (the kinetic merical number derived out of the power mechanics profile of PM concentration as a function of VLTI time) for four cycles were ascertained as 0.0177 min^−1^, 0.0186 min^−1^, 0.0164 min^−1^, and 0.0151 min^−1^. Additionally, *K_TOC_* (the kinetic merical number derived through the power mechanics profile of TOC concentration and VLTI time) for four cycles were detected as 0.0131 min^−1^, 0.0140 min^−1^, 0.0161 min^−1^, and 0.0131 min^−1^. These results indicate that the photodegradation of PM in pesticide wastewater using SZHP follows the kinetics characterized by first-order kinetics.

**Figure 16:**
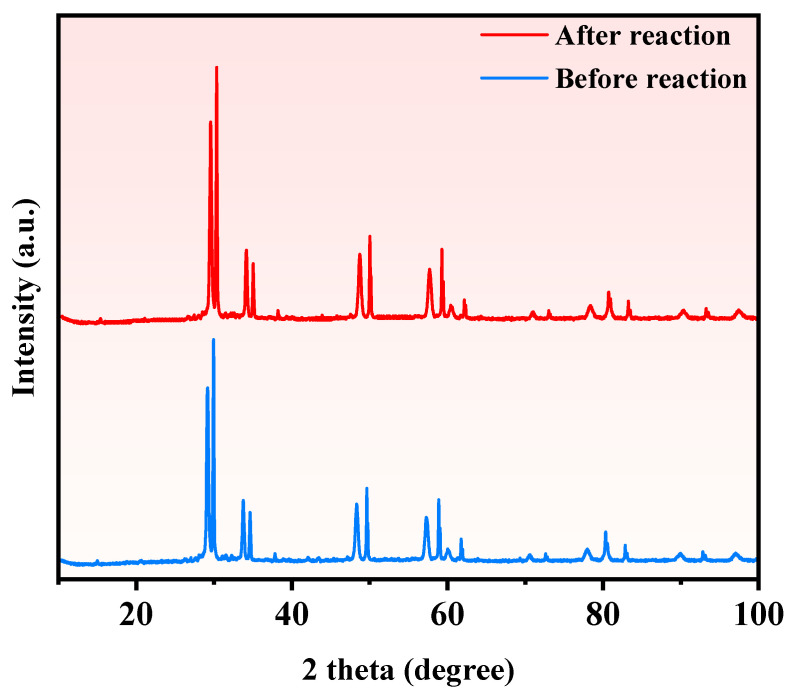
The XRD pattern of the sustained recyclable performance of PDD of PM with SZHP in the function of a photocatalytic material.

**Figure 17:**
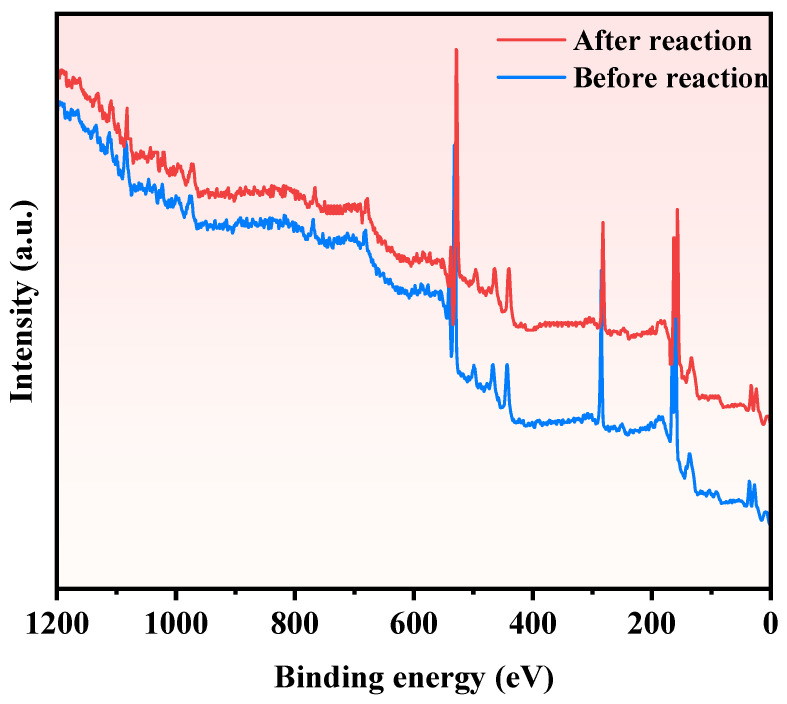
The XPS spectrum of the sustained recyclable performance of PDD of PM with SZHP in the function of a photocatalytic material.

Figure 16 showcases the XRD pattern of the synthesized SZHP materials before and after PDD of PM. The XRD analysis confirms the preservation of the crystal structure of SZHP, indicating no significant alterations throughout the PDD process. Additionally, Figure 17 presents the XPS pattern of SZHP materials before and after the PDD of PM. The XPS analysis affirms that no noteworthy changes occurred in the elemental content, molecular structure, and atomic valence states of elements of SZHP following the PDD process. Therefore, it can be inferred that the shape and position of the high-resolution lines will likewise not change. In summary, both the XRD and XPS analyses consistently demonstrate the structural integrity of SZHP throughout the PDD of PM. These findings collectively underscore the robustness and chemical stability of SZHP during the photocatalytic treatment of PM.

**Figure 18:**
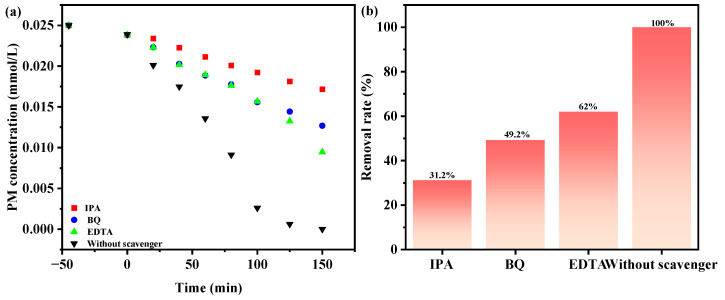
The implication of various radical interceptors on (**a**) PM concentration and (**b**) elimination efficiency of PM with SZHP in the function of a photocatalytic material under VLTI.

Figure 18 investigates the effect of diverse radical scavengers, including benzoquinone (BQ), isopropanol (IPA), and ethylenediamine tetraacetic acid (EDTA), on the degradation rate of PM using SZHP under visible light exposure [111]. These scavengers were introduced at the initiation of the PDD experiments to investigate the active intermediates participating in the PDD course. Hydroxyl radicals (•OH) were apprehended through the integration of IPA. BQ was utilized to apprehend superoxide anions (•O_2_^−^), which were apprehended through the integration of BQ. Apprehended holes (h^+^) were apprehended through the integration of EDTA. The formulary IPA saturation or BQ saturation or EDTA saturation was 0.10 mmol L^−1^, and the input quantity of IPA or BQ or EDTA was 1.5 mL. With the existence of IPA, BQ, or EDTA, the decomposition velocity of PM decreased by 68.8%, 50.8%, and 38%, respectively, compared with the standard group. These findings propose the involvement of ·OH, ·O_2_^−^ and h^+^ as active radicals in the PDD process of PM. Hydroxyl radicals exhibited the highest oxidation removal ability in eliminating PM in pesticide wastewater when using SZHP as a light-activated photocatalytic material. The order of oxidation removal ability for degrading PM is as follows, in descending order: ·OH> ·O_2_^−^> h^+^.

**Figure 19:**
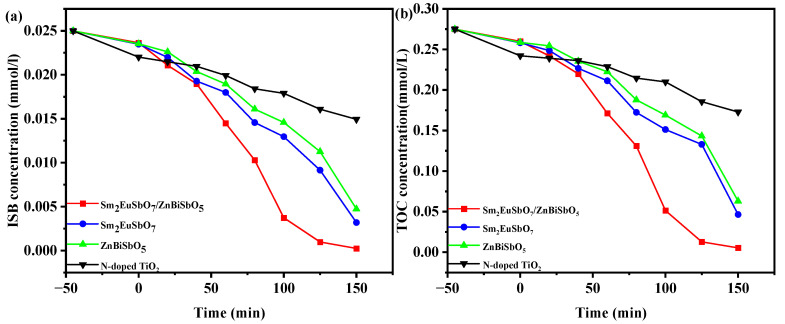
Saturation variation profiles of (**a**) ISB and (**b**) TOC during the photodegradation of ISB with SZHP, Sm_2_EuSbO_7_, ZnBiSbO_5_, or N-TO as the photocatalyst under VLTI.

For the purpose of verifying the pivotal significance of fabricated photocatalysts in practical water pollution control projects, another widely used insecticide, isocarbophos (ISB), was used as the target pollutant in the degradation experiments. The photocatalysts employed in this investigation consisted of SZHP, Sm_2_EuSbO_7_, ZnBiSbO_5_, and N-TO. The concentration patterns of ISB in pesticide wastewater over time under VLTI are depicted in Figure 19a. The obtained results clearly demonstrate a consistent decline in the concentration of ISB corresponding to the extended period of irradiation.

Analysis of the data obtained from Figure 19a reveals that the use of SZHP as the photocatalyst resulted in complete removal of ISB within pesticide wastewater, achieving a reaction velocity of 2.76 × 10^−9^ mol·L^−1^·s^−1^ and PNE of 0.0580% after 150 min of VLTI. The other photocatalysts were subjected to similar experimental conditions. When Sm_2_EuSbO_7_ was utilized as the photocatalyst, it achieved a remarkable ISB removal rate of 87.2%, demonstrating a reaction velocity of 2.42 × 10^−9^ mol·L^−1^·s^−1^ and PNE of 0.0509%. Likewise, in the case of ZnBiSbO_5_ as the photocatalyst, a significant ISB removal rate of 81.2% was achieved in pesticide wastewater, accompanied by a reaction velocity of 2.26 × 10^−9^ mol·L^−1^·s^−1^ and PNE of 0.0474%. Lastly, N-TO exhibited an ISB removal rate of 40.4%, indicating a reaction velocity of 1.12 × 10^−9^ mol·L^−1^·s^−1^ and PNE of 0.0236%.

As indicated by the above evidence, it can be inferred that SZHP demonstrated the highest photodegradation efficiency for ISB compared with the other photocatalysts. Additionally, the photodegradation efficiency achieved using Sm_2_EuSbO_7_ as the photocatalyst was superior to that of ZnBiSbO_5_ and N-TO. Furthermore, the photodegradation efficiency obtained with ZnBiSbO_5_ as the photocatalyst surpassed that of N-TO. These findings indicate that SZHP exhibits enhanced photocatalytic activity under VLTI compared with individual Sm_2_EuSbO_7_, ZnBiSbO_5_, and N-TO materials.

Moreover, upon comparing the ISB degradation velocity achieved through 150 min of VLTI, it becomes clear that the effectiveness of SZHP surpassed that of Sm_2_EuSbO_7_ by 1.15 times, ZnBiSbO_5_ by 1.23 times, and N-TO by 2.48 times.

The saturation variation profiles of TOC throughout the PDD of ISB in pesticide effluent under VLTI using different photocatalysts are presented in Figure 19b. The investigated photocatalysts include SZHP, Sm_2_EuSbO_7_, ZnBiSbO_5_, and N-TO. As illustrated in Figure 17b, the TOC concentration exhibited reduction as the VLTI time increased.

Analysis of Figure 19b demonstrates that following a 150-min duration of VLTI, the degradation rates of TOC within pesticide wastewater were found to be 98.04%, 88.13%, 77.09%, and 37.16% when employing SZHP, Sm_2_EuSbO_7_, ZnBiSbO_5_, and N-TO as photocatalysts, respectively, for the treatment of ISB.

**Figure 20:**
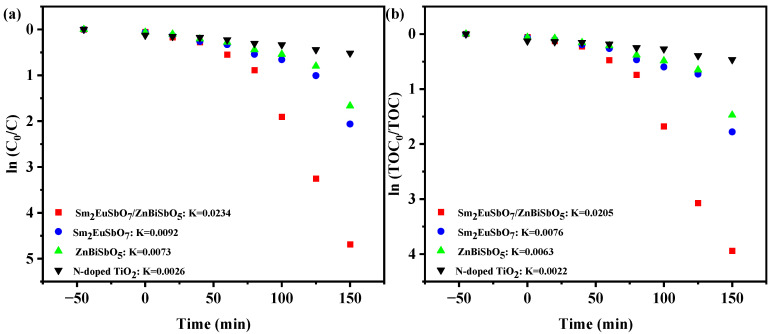
Identified first-order kinetics charts for (**a**) ISB and (**b**) TOC during the photodegradation of ISB with SZHP, Sm_2_EuSbO_7_, ZnBiSbO_5_, or N-TO as the photocatalyst under VLTI.

Figure 20 exhibits the first-order kinetic schematics of ISB (Figure 20a) and TOC (Figure 20b) during the photodegradation of ISB with various photocatalysts, including SZHP, Sm_2_EuSbO_7_, ZnBiSbO_5_, and N-TO, under VLTI. *K_C_* (the kinetic merical number derived through the power mechanics profile of ISB saturation and VLTI time) were 0.0234 min^−1^, 0.0092 min^−1^, 0.0073 min^−1^, and 0.0026 min^−1^ for SZHP, Sm_2_EuSbO_7_, ZnBiSbO_5_, and N-TO, respectively. Additionally, *K_TOC_* (the kinetic merical number derived through the power mechanics profile of TOC saturation as a function of VLTI time) were ascertained as 0.02005 min^−1^, 0.0076 min^−1^, 0.0063 min^−1^, and 0.0022 min^−1^ for SZHP, Sm_2_EuSbO_7_, ZnBiSbO_5_, and N-TO, respectively. Notably, *K_TOC_* was lower than *K_C_* for all the catalysts, indicating the formation of intermediate species during the PDD process. SZHP displayed superior mineralization efficiency for ISB degradation in relation to other catalysts being evaluated.

**Figure 21:**
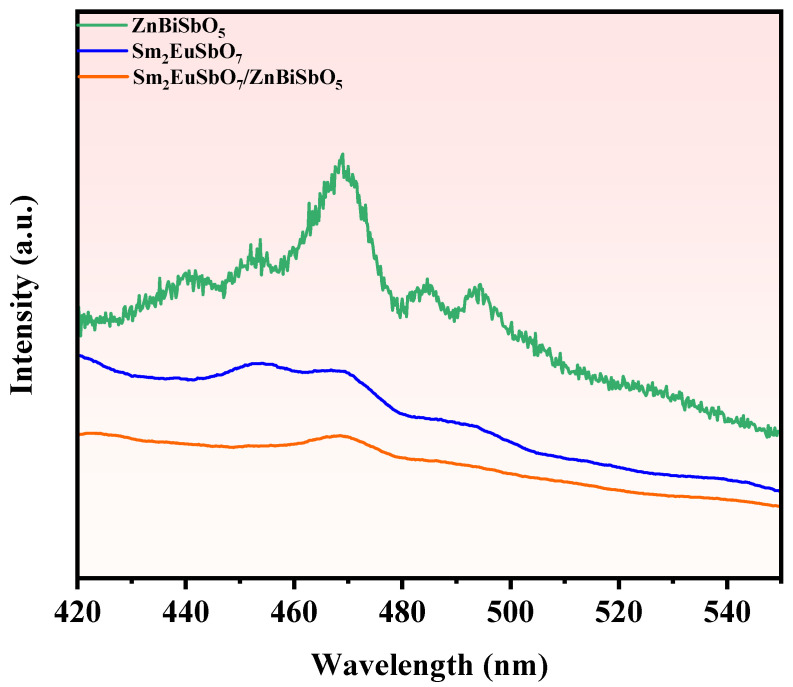
PL spectrum of SZHP, Sm_2_EuSbO_7_, and ZnBiSbO_5_.

Photoluminescence (PL) spectroscopy is an invaluable method for examining the migration, spatial segregation, and recombination kinetics of photoexcited carriers. Higher PL intensities typically indicate faster recombination rates of the photogenerated carriers, leading to reduced photocatalytic efficiency [112,113]. Figure 21 illustrates the PL spectrum of SZHP, prepared via the solvothermal method, as well as Sm_2_EuSbO_7_ and ZnBiSbO_5_ prepared through the solid-state calcination method. Based on Figure 21, it is evident that the ZnBiSbO_5_ sample displays the highest emission intensity, suggesting significant recombination of the photoexcited carriers. Comparatively, the Sm_2_EuSbO_7_ sample displays a lower emission intensity compared with ZnBiSbO_5_, while SZHP exhibits an even lower emission intensity relative to both Sm_2_EuSbO_7_ and ZnBiSbO_5_. This observation suggests a pronounced enhancement in the spatial isolation of photoexcited carriers through the formation of the heterojunction. Furthermore, this provides further evidence that SZHP showcases the superior photocatalytic efficiency among Sm_2_EuSbO_7_ and ZnBiSbO_5_ samples.

**Figure 22:**
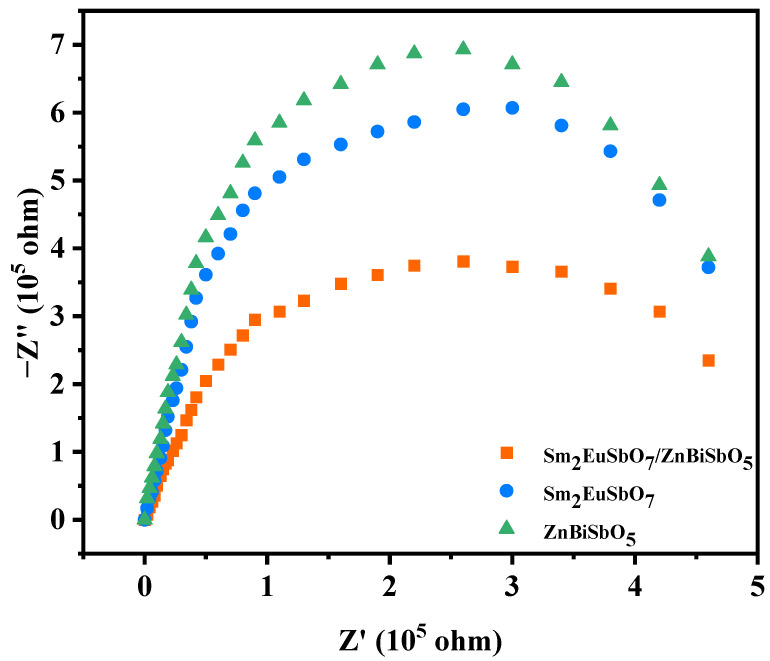
Nyquist impedance plots of SZHP, Sm_2_EuSbO_7_, and ZnBiSbO_5_.

The electrochemical impedance spectroscopy (EIS) is a significant characterization technique that provides insights into the migration process of photogenerated carriers at the interfaces of two single-crystal photocatalysts forming a heterojunction [114,115]. In this study, the impedance arc size observed in the Nyquist impedance plot was utilized to evaluate the migration efficiency of the prepared photocatalysts, where a smaller arc radius indicates superior efficiency. Figure 22 presents the EIS of Sm_2_EuSbO_7_ and ZnBiSbO_5_, both synthesized through solid-phase sintering, along with SZHP, which was prepared using the solvothermal method. Examination of Figure 18 reveals a distinct trend in the arc radius diameter: ZnBiSbO_5_ > Sm_2_EuSbO_7_ > SZHP. This observation suggests that the SZHP photocatalysts possess enhanced photocatalytic activity by separating photoexcited carriers more efficiently and enhancing the interface charge mobility at the same time compared with the Sm_2_EuSbO_7_ and ZnBiSbO_5_ photocatalysts. The results of this analysis are exactly consistent with those of Figure 12, Figure 13, Figure 19 and Figure 21 [116].

### 2.7. Analysis of Possible Degradation Mechanisms

**Figure 23:**
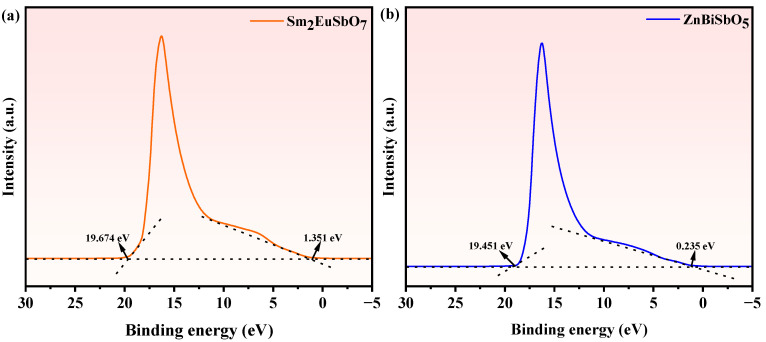
Ultraviolet photoelectron spectrum (UPS) of (**a**) Sm_2_EuSbO_7_ and (**b**) ZnBiSbO_5_.

**Figure 24:**
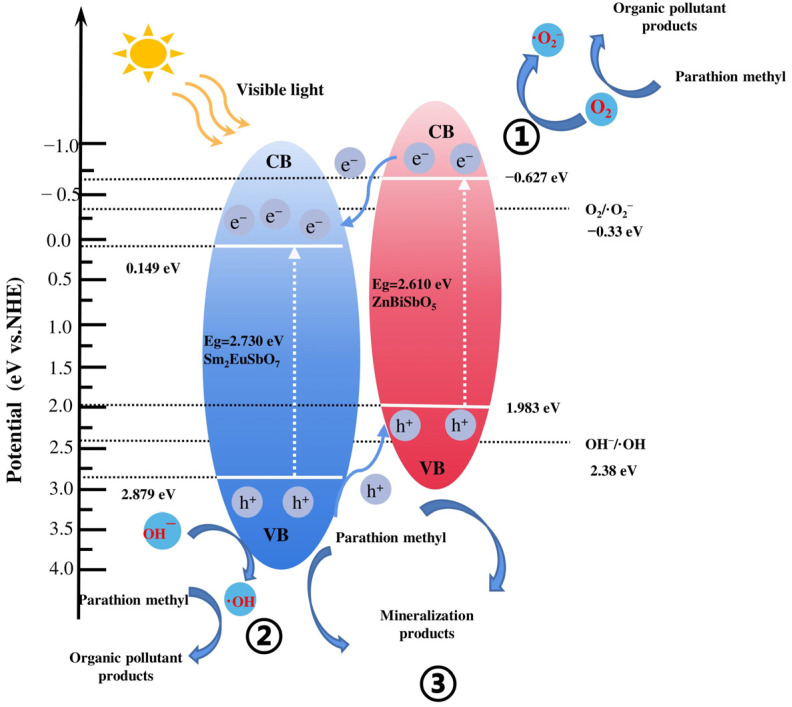
Possible photodegradation mechanism of PM with SZHP as photocatalyst under VLTI.

Figure 23 showcases the Ultraviolet photoelectron spectrum (UPS) of Sm_2_EuSbO_7_ and ZnBiSbO_5_. And the possible PDD mechanistic model of PM with SBHP under VLTI is showcased in Figure 24. The potentials of the valence band (VB) and conductor band (CB) for light-activated materials can be determined using Equations (3) and (4) [117]:

*E*_CB_ = *X* − *E*^e^ − 0.5*E*_g_ (3)

*E*_VB_ = *E*_CB_ + *E*_g_ (4)

where, *E*_g_ was the band gap of catalyst, *X* was the electronegativity of the catalyst, and *E*^e^ was the energies of the hydrogen-like scale of free electrons (close to the 4.5 eV mark). The VB potential or the CB potential for Sm_2_EuSbO_7_ was approximated as 2.879 eV or 0.149 eV severally. For ZnBiSbO_5_, the VB potential or the CB potential was approximated as 1.983 eV and −0.627 eV severally. Both Sm_2_EuSbO_7_ and ZnBiSbO_5_ exhibited light absorption in the visible portion, leading to the generation of photoexcited carriers.

To strengthen the credibility of the mechanistic model, ultraviolet photoelectron spectroscopy (UPS) was conducted to assess the ionization potential of Sm_2_EuSbO_7_ and ZnBiSbO_5_. The acquired UPS spectra exhibiting the onset (Ei) and cutoff (Ecutoff) binding energies for both photocatalysts were featured in Figure 23, measuring at 1.351 eV and 19.674 eV for Sm_2_EuSbO_7_, and 0.235 eV and 19.451 eV for ZnBiSbO_5_. Calculating from the excitation energy (approximately 21.2 eV), the ionization potential of Sm_2_EuSbO_7_ or ZnBiSbO_5_ was determined to be 2.877 eV or 1.984 eV severally. Consequently, the CB potential of Sm_2_EuSbO_7_ or ZnBiSbO_5_ was measured to be 0.147 eV or −0.626 eV, respectively, which closely aligned with the numerical figures derived from Equations (3) and (4).

On account of the more negative redox potential status of the CB of ZnBiSbO_5_ (−0.627 eV) compared with that of the Sm_2_EuSbO_7_ (0.149 eV), light-induced carriers on the CB of ZnBiSbO_5_ could be transmitted to the CB of Sm_2_EuSbO_7_. Similarly, on account of the elevated redox potential position of the VB of Sm_2_EuSbO_7_ (2.879 eV) compared with that of ZnBiSbO5 (1.983 eV), light-induced carriers on the VB of Sm_2_EuSbO_7_ could be transmitted to the VB of ZnBiSbO5. The integration of Sm_2_EuSbO_7_ and ZnBiSbO_5_ in SZHP successfully mitigated the recombination velocity of photoinduced carriers. This reduction resulted in decreased internal resistance, longer carrier lifetimes, and improved interfacial charge transfer efficiency. Consequently, the production of active species such as ·OH or ·O_2_^−^ was increased, leading to an improved degradation velocity of PM.

Additionally, this study speculated on the possible mechanisms for the generation of ·OH and ·O_2_^−^ [118,119]. The CB potential of ZnBiSbO_5_ (−0.627 eV) was lower on the negative scale than that of O_2_/·O_2_^−^ (−0.33 eV vs. NHE), signifying that the carriers within the CB of ZnBiSbO_5_ could consume oxygen to generate ·O_2_^−^, which can degrade PM (① in Figure 24) [120]. Similarly, the VB potential of Sm_2_EuSbO_7_ (2.879 eV) was higher on the positive scale than the magnitude of OH^−^/·OH (2.38 eV vs. NHE), signaling that the carriers in the VB of Sm_2_EuSbO_7_ could transform H_2_O or OH^−^ into ·OH for PM degradation (② in Figure 24) [121,122]. Ultimately, the light-activated positive carriers in the VB of ZnBiSbO_5_ or Sm_2_EuSbO_7_ could directly catalyze the oxidation and subsequent degradation of particulate matter (PM) owing to their inherent robust oxidation efficiency (③ in Figure 24). Overall, the exceptional photocatalytic efficiency of SZHP in degrading PM can be primarily attributed to its remarkable efficiency in separating carriers within the photocatalytic material.

**Figure 25:**
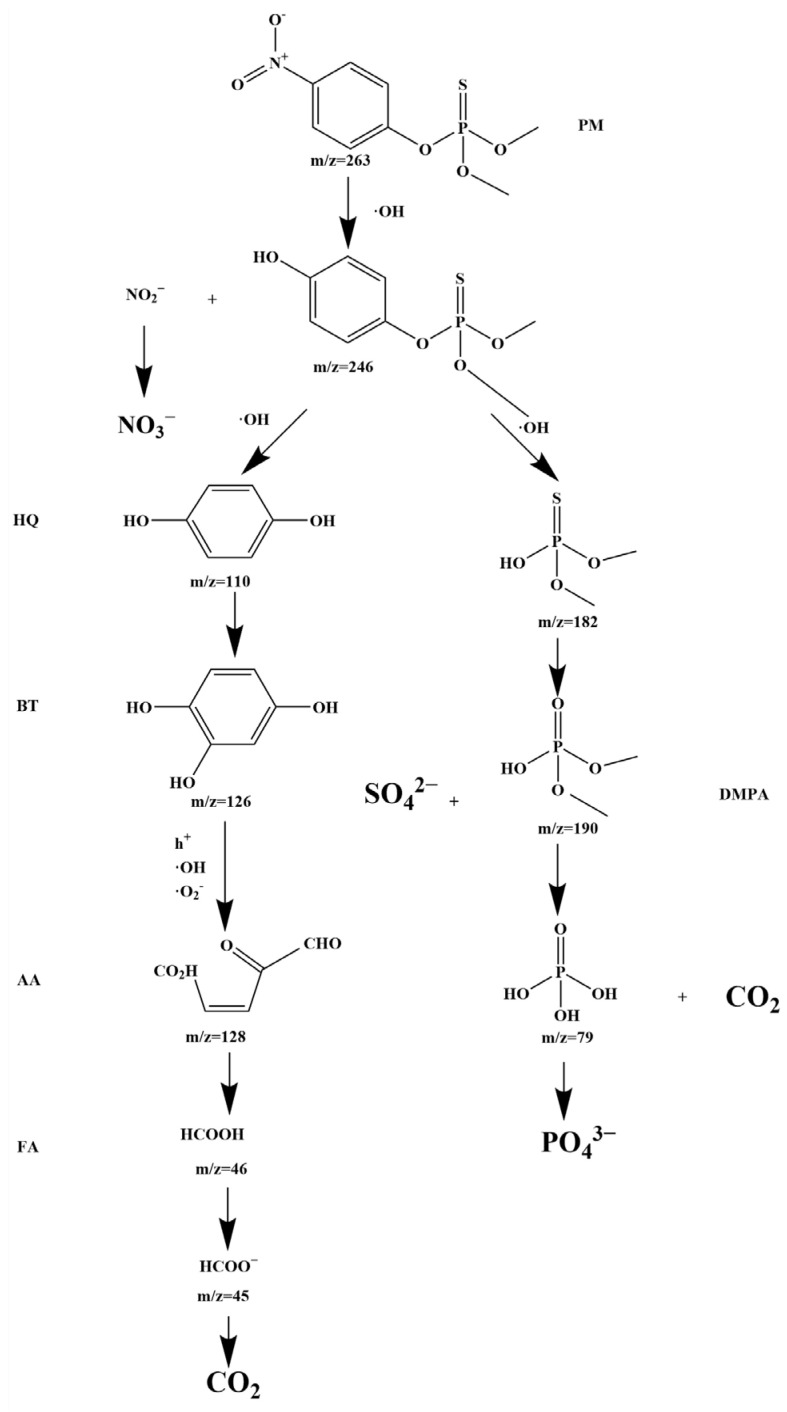
Proposed PDD pathway diagram for PM with SZHP in the function of a photocatalytic material under VLTI.

With the objective of analyzing the degradation mechanism of PM, the intermediates produced during PM degradation were examined using the LC-MS method. The degradation of PM occurs through the following pathways, as shown in Figure 25. The nitro-phenyl bond in PM (*m*/*z* = 263) is directly oxidized by a hydroxyl radical (·OH), resulting in the formation of C_6_H_4_OPS(OCH_3_)_2_OH (*m*/*z* = 246) and nitrite ions (NO_2_·O^2−^). Nitrogen enters the dissolving fluid in the form of nitrite ions (NO_2_·O^2−^) and is eventually oxidized to more stable nitrate ions (NO_3_^−^). The P-O bond connecting to the aromatic ring is cleaved, producing the highly unstable intermediate PS(OCH_3_)_2_OH (*m*/*z* = 182), which further transforms into dimethyl phosphate (DMPA), along with the formation of sulfate ions (SO_4_^2−^). DMPA (*m*/*z* = 190) continues to degrade under the strong oxidative power of hydroxyl radicals (·OH), producing phosphate ions (PO_4_^3−^) and carbon dioxide (CO_2_) as the final products. Meanwhile, the degradation process generates and consumes hydroquinone (HQ) (*m*/*z* = 110) and 1,2,4-benzenetriol (BT) (*m*/*z* = 126). Under the action of multiple active free radicals (h^+^, ·O^2−^, ·OH) aromatic compounds are degraded to produce fatty acids, which further transform into aliphatic acid (AA) (*m*/*z* = 128). Subsequently, AA is further decomposed into formic acid (FA) (*m*/*z* = 46), formate ions (*m*/*z* = 45), and carbon dioxide (CO_2_) under the action of hydroxyl radicals.

## 3. Experimental Section

### 3.1. Materials and Reagents

Ethylenediaminetetraacetic acid (EDTA, C_10_H_16_N_2_O_8_, purity level = 99.5%) and isopropyl alcohol (IPA, C_3_H_8_O, purity level ≥ 99.7%) were analytical grade. P-benzoquinone (BQ, C_6_H_4_O_2_, purity level ≥ 98.0%) was laboratory grade. Absolute ethanol (C_2_H_5_OH, purity level ≥ 99.5%) and PM (C_8_H_10_NO_5_PS, purity level ≥ 99.0%) was gas chromatography grade. The aforementioned chemical solutions were procured from Aladdin Group Chemical Reagent Co., Ltd. (Shanghai, China). Ultra-pure water with a resistivity of 18.25 MU cm was utilized consistently from the beginning to the end of the experimental procedures.

### 3.2. Fabrication Method of ZnBiSbO_5_

ZnBiSbO_5_ was fabricated through the solid-state calcination method conducted at an elevated temperature. ZnO (99.999%), Bi_2_O_3_ (99.999%), and Sb_2_O_5_ (99.995%) were used as raw materials, obtained from Aladdin Group Chemical Reagent Co., Ltd. (Shanghai, China). To determine the molar ratio of the raw oxides, volatility tests were conducted as follows: 0.02 mol ZnO, 0.01 mol Bi_2_O_3_, and 0.01 mol Sb_2_O_5_ were individually measured and pulverized for 3 h in an agate mortar. The ground oxides were individually positioned in an alumina crucible (Shenyang Crucible Co., Ltd., Shenyang, China) and calcined at 1300 °C for 25 h in a high-temperature electric furnace (KSL 1700X, Hefei Kejing Materials Technology Co., Ltd., Hefei, China). After being scorified at 400 °C for 2 h, the untreated materials and diminutive columns were removed from the electric stove. The mixed materials were then ground and placed in a heat treatment furnace for calcination at 1300 °C for 20 h. After naturally reaching room temperature, the mass of ZnO, Bi_2_O_3_, and Sb_2_O_5_ was measured and converted back to moles. The results indicated that 0.02 mol ZnO and 0.01 mol Sb_2_O_5_ exhibited no variation, while 0.008 mol Bi_2_O_3_ was left, suggesting its volatility at 1300 °C. Through multiple failed experiments, it was determined that 20% of Bi_2_O_3_ would volatilize, and thus the practical mole quantity dispensing of Bi_2_O_3_ should have been 120% of the planned dosage.

The fully mixed materials were quantified based on stoichiometry (n(ZnO): n(Bi_2_O_3_): n(Sb_2_O_5_) = 2:1.2:1) and placed into a ball grinder to achieve a lattermost particle dimension of 0.5–1.5 µm. In the cause of achieving a high level of purity, the powders underwent a pre-synthesis drying step at 200 °C for 4 h. Subsequently, the dried dusty materials were combined in an aluminum oxide crucible, compressed into a circular tray, and calcined in a heat treatment furnace at 420 °C for 7.8 h. Following the process of pulverizing and pasting, the mixture underwent additional sintering in a heat treatment furnace at 1300 °C for a period of 20 h. Ultimately, the pure ZnBiSbO_5_ catalyst was acquired via the process of thorough pulverizing.

Hydrothermal synthesis offers several advantages, including high purity, excellent dispersion, and ease of controlling particle size. These attributes make it an ideal method for controlling the size and morphology of the final product. In line with this, the present study outlined the experimental procedures which were used for fabricating ZnBiSbO_5_ through hydrothermal synthesis. For the synthesis of ZnBiSbO_5_, 0.11 mol/L Zn(NO_3_)_2_·5H_2_O, 0.11 mol/L Bi(NO_3_)_3_·5H_2_O, and 0.11 mol/L SbCl_5_ were combined and subjected to stirring for 20 h. The dissolving fluid was then transferred to a Teflon-lined autoclave and heated at 200 °C for 15 h. The resulting powder was further calcined at 780 °C for 10 h in a tube furnace under an atmosphere of N_2_.

### 3.3. Fabrication Method of Sm_2_EuSbO_7_

The Sm_2_EuSbO_7_ photocatalytic material was fabricated using the solid-state calcination method. Sm_2_O_3_, Eu_2_O_3_, and Sb_2_O_5_ with purities of 99.999%, which were used as ingredients, were attained from Aladdin Group Chemical Reagent Co., Ltd. (Shanghai, China). The catalytic material was fabricated by drying ingredients at 200 °C for 2 h in a ratio of n(Sm_2_O_3_):n(Eu_2_O_3_):n(Sb_2_O_5_) = 2:1:1. A stoichiometric blend of the precursor was then prepared, which was compressed into a diminutive column and placed in an alumina crucible from Shenyang Crucible Co., Ltd. (Shenyang, China). After heating at 500 °C for 3.5 h, the primary material and diminutive cylinder were extracted from the heat treatment furnace. The ingredients were pulverized and subsequently positioned in the electric furnace (KSL 1700X, Hefei Kejing Materials Technology Co., Ltd., Hefei, China). Finally, the components were subjected to 1100 °C calcination for 25 h using an electric smelter.

The experimental procedures for the fabrication of Sm_2_EuSbO_7_ using the hydrothermal fabricate method are described as follows: 0.22 mol/L Sm(NO_3_)_3_·6H_2_O, 0.11 mol/L Eu(NO_3_)_3_·6H_2_O, and 0.11 mol/L SbCl_5_ were agitated and mixed for 20 h. The solvent system was then transported over to a Teflon-lined autoclave, and the temperature was raised to 400 °C for 8 h. The resulting catalytic material was thermally processed at 750 °C for 11 h in the tube furnace with the atmosphere condition of N_2_.

### 3.4. Fabrication of N-Doped TiO_2_

In this study, N-TO catalytic material was synthesized using the solution-gel technique. Tetrabutyl titanate was employed as the precursor and ethanol served as the solvent. The experimental procedure was carried out in the following steps:

Initially, a solution A was prepared by combining 15 mL of tetrabutyl titanate with 38 mL of absolute ethyl alcohol. Subsequently, a solution B was prepared by mixing 38 mL of absolute ethyl alcohol, 8 mL of glacial acetic acid, and 3 mL of double distilled water. The resultant solution α was then drip fed into solution β while vigorously stirring with a magnetic stirrer, leading to the formation of a transparent colloidal suspension.

To introduce nitrogen into the system, an appropriate amount of N/Ti ratio of 8 mol% ammonia was added to the gel solvent system with transparency and magnetic rotational mixing was continued for 2 h. Following this, the mixture was allowed to age for 48 h, resulting in the formation of aluminum hydroxide gel. The aluminum hydroxide gel was subsequently ground into a fine powder and subjected to calcination at a temperature of 500 °C for a duration of 2 h. Finally, the resulting powder was further refined by passing it through a vibrating screen, yielding the N-TO powder. This nitrogen-doped titania catalyst was utilized for subsequent investigations.

### 3.5. Fabrication of Sm_2_EuSbO_7_/ZnBiSbO_5_ Heterojunction Photocatalyst

ZnBiSbO_5_ and Sm_2_EuSbO_7_ were fabricated via the solid-state calcination method, with maximum calcination temperatures of 1300 °C and 1100 °C, respectively, and durations of heat treatment of 20 h and 25 h, respectively. The hydrothermal fabricate method was also utilized for the fabrication of ZnBiSbO_5_ and Sm_2_EuSbO_7_, with maximum calcination temperatures of 880 °C and 750 °C, respectively, and a duration of 13 h for both samples.

It is important to consider that higher calcination temperatures lead to elevated power energy consumption, which can potentially diminish the lifespan of the furnace instrument. Additionally, longer insulated times and higher calcination temperatures led to larger particle sizes, resulting in reduced specific surface areas and photocatalyst activity of Sm_2_EuSbO_7_ and ZnBiSbO_5_.

To boost the photocatalyst’s efficiency, minimize energy wastage, and prolong the lifespan of the high-temperature calcining furnace, the hydrothermal fabricate method was chosen to fabricate Sm_2_EuSbO_7_ and ZnBiSbO_5_ in the heterojunction process.

The hydrothermal synthesis method involved the dissolving reformation process, where Sm(NO_3_)_3_·6H_2_O, Eu(NO_3_)_3_·6H_2_O, SbCl_5_, Zn(NO_3_)_2_·5H_2_O, and Bi(NO_3_)_3_·5H_2_O were completely dispersed in a hydrothermal medium. The resulting ion and molecular groups were transported to the growth area with seed crystals due to strong convection resulting from temperature variation in the autoclave. Ultimately, an oversaturated solution was established and experienced crystallization.

For the synthesis of ZnBiSbO_5_, 0.11 mol/L Zn(NO_3_)_2_·5H_2_O, 0.11 mol/L Bi(NO_3_)_3_·5H_2_O, and 0.11 mol/L SbCl_5_ were combined and subjected to stirring for 20 h. The dissolving fluid was then transferred to a Teflon-lined autoclave and heated at 200 °C for 15 h. The resulting powder was further calcined at 780 °C for 10 h in a tube furnace under an atmosphere of N_2_. For the synthesis of Sm_2_EuSbO_7_, 0.22 mol/L Sm(NO_3_)_3_·6H_2_O, 0.11 mol/L Eu(NO_3_)_3_·6H_2_O, and 0.11 mol/L SbCl_5_ were agitated and mixed for 20 h. The solution was then transferred to a Teflon-lined autoclave and heated at 400 °C for 8 h. The resulting catalytic material was calcined at 750 °C for 11 h in the tube furnace with the atmosphere condition of N_2_.

The solvothermal technique was employed to dissolve the powders of Sm_2_EuSbO_7_ and ZnBiSbO_5_ in an octanol organic solvent within an autoclave. Operating under the conditions of liquid phase and supercriticality, the reactants became highly reactive and dispersed, resulting in a gradual synthesis of the desired product.

The solvothermal method was employed to synthesize a novel SZHP by combining ZnBiSbO_5_ and Sm_2_EuSbO_7_ in octanol. The mixture underwent ultrasonic treatment, followed by heating and stirring conditions. This process aimed to facilitate the adhesion of ZnBiSbO_5_ onto the surface of Sm_2_EuSbO_7_ nanoparticles, resulting in the emergence of SZHP. The resulting product was purified, dried, and stored for further use.

Moreover, based on the above preparation method, the approximate costs of N-TO, ZnBiSbO_5_, Sm_2_EuSbO_7_, and SZHP were about $365.82, $25.22, $20.61, and $22.48, respectively. It is apparent that the cost of N-TO was 15.51 times higher than that of ZnBiSbO_5_, 17.75 times higher than that of Sm_2_EuSbO_7_, and 16.27 times higher than that of SZHP. To evaluate the potential toxicity of the ZnBiSbO_5_ photocatalyst, Sm_2_EuSbO_7_ photocatalyst, and SZHP, phytotoxicity tests were conducted using wheat as a target plant. The tests measured the germination rate of wheat after exposure to the aforementioned photocatalysts. The results indicated that wheat exhibited a 100% germination rate within a period of 9 days. Moreover, to investigate the potential toxicity of individual elements present in the photocatalysts, a comprehensive assessment was carried out to detect the presence of toxic metals, such as antimony, in the solution after pollutant degradation. The findings revealed the absence of residual toxic metal elements in the solution, suggesting the highly efficient elimination of toxic metals during the pollutant degradation process. This outcome further supported the eco-friendly nature of ZnBiSbO_5_, Sm_2_EuSbO_7_, and SZHP photocatalysts, underscoring their suitability for potential applications in pollutant remediation.

### 3.6. Characterization

In this article, various characterization technologies including XRD, TEM, XPS, FTIR spectrometer, Raman spectrometer, fluorescence spectrophotometer, and UV-Vis diffuse reflectance spectrophotometer (UV-VIS-DRS) were employed for elucidating the anatomical features of pure-phase Sm_2_EuSbO_7_ and pure-phase ZnBiSbO_5_ which were fabricated utilizing the power-controlled solid-phase calcination method. Additionally, the decomposition rate of PM under VLTI with pure-phase Sm_2_EuSbO_7_, pure-phase ZnBiSbO_5_, N-TO, or SZHP as the presence of the catalyst was identified.

The diffraction patterns of the fabricated materials were analyzed by XRD (Shimadzu, XRD-6000, Cu Kα radiation, λ = 1.54184 Å, sampling pitch of 0.02°, preset time of 0.3 s step^−1^, Kyoto, Japan). The external and microscopic characteristics of the fabricated samples were determined using TEM (JEM—F200 FEI Tecnai G2 F20 FEI Talos F200s) and the elemental constituents were measured by EDS. A diffuse reflectance spectrum of the fabricated materials was acquired employing UV-VIS-DRS (Shimadzu, UV-3600, Kyoto, Japan). The functional groups and chemical bonds were analyzed by FTIR (WQF-530A, Beifen-Ruili Analytical Instrument (Group) Co., Ltd., Beijing, China). The interactions among the various chemical bonds were analyzed with a Raman spectrometer (INVIA0919-06, RENISHAW plx, Wotton-under-Edge, Gloucestershire, UK). The elemental constituents and surface chemical states of the fabricated sample were assessed by XPS (Escalab 250Xi, Thermo Fisher Scientific Corporation, Waltham, MA, USA) with an Al-kα X-ray source.

### 3.7. Photoelectrochemical Experiments

Electrochemical impedance spectroscopy (EIS) was acquired with the assistance of a CHI660D electrochemical station (Chenhua Instruments Co., Ltd., Shanghai, China) with a typical three-electrode configuration. The working electrode (as-fabricated materials), counter electrode (platinum plate), and reference electrode (Ag/AgCl) formed three parts of the three electrodes system, and the electrolyte fluid was an Na_2_SO_4_ watery solution (0.5 mol/L). The light simulation for the experiment was conducted through a 500 W Xe lamp with a 420 nm cut-off filter. The manufacture of the working electrode was as follows: 8 mg of fabricated materials was dispersed in a mixture of 640 μL of ethanol, 960 μL of ultrapure water, and 32 μL Nafion solution and sonicated for 50 min. Then, 8 μL of the suspension was taken and evenly dripped on the surface of the polished glass carbon electrode, and put under the infrared light (that is, the working electrode) to dry.

### 3.8. Experimental Setup and Procedure

The PDD experiment was conducted using a photocatalytic reactor (CEL-LB70, China Education Au-Light Technology Co., Ltd, Beijing, China) at a constant temperature of 20 °C, which was regulated by recirculating cool water. Solar radiation simulation was supplied by a 500 W xenon lamp with a 420 nm cut-off filter.

In each experiment, 12 quartz tubes were used, with a volume of 55 mL for a single reaction solution. For chemical industry wastewater, the total experimental solution volume was 660 mL. The dosage of the catalysts (Sm_2_EuSbO_7_ or ZnBiSbO_5_ or SZHP) was 0.75 g/L. The initial saturation of parathion methyl was 0.040 mmol/L, which represented the remaining saturation after biodegradation of pesticide wastewater that originally contained a parathion methyl concentration of 1.2 mmol/L.

Throughout the reaction time frame, a 3 mL dissolving fluid was extracted for analysis at regular intervals, and a 27 mL dissolving fluid was retrieved after VLTI-150 min to measure the saturation of residual PM. The catalyst was subsequently separated by filtration employing a filter membrane made of 0.22 µm PES polyether sulfone. The remaining saturation of PM in the solvent system was assessed using Agilent 200 high-performance liquid chromatography (Agilent Technologies, Palo Alto, CA, USA) equipped with a UV detector and a Zorbax 300SB-C18 column (4.6 mm × 150 mm, 5 μm) for analysis. A mixture of 50% acetonitrile and 50% distilled water made up the moving phase, and the UV measuring wavelength was set at 254 nm. The shot capacity of the PDD parathion methyl solution was 10 μL and the current velocity was set at 1 mL/min^−1^.

To achieve adsorption/desorption equilibrium between the photocatalyst, PM, and atmospheric oxygen, the dissolving fluid containing the photocatalyst and PM was stirred under darkness through magnetic rotational mixing conditions for 45 min prior to exposure to VLTI. The homogeneous mixture was stirred at a speed of 500 rpm.

The TOC analyzer (TOC-5000 A, Shimadzu Corporation, Kyoto, Japan) was employed to quantify the mineralization experimental data of parathion methyl within the reaction solution. To determine the saturation of TOC during the PDD of parathion methyl, potassium acid phthalate (KHC_8_H_4_O_4_) was employed as a certified reference material. For calibration purposes, a standard solvent system of potassium acid phthalate with varying carbon content from 0 to 100 mg/L was prepared. The TOC saturation was measured in six samples, with each sample consisting of 45 mL of the solvent system.

PM and its PDD products were analyzed and quantified using liquid chromatography–mass spectrometry (LC–MS) on a Thermo Quest LCQ Duo system (Thermo Fisher Scientific Corporation, Waltham, MA, USA). Separation was carried out on a Beta Basic-C18 HPLC column (150 × 2.1 mm, 5 μm ID, Thermo Fisher Scientific Corporation, Waltham, MA, USA). A 20 μL sample obtained from the PDD reaction was injected into the LC-MS system. The flowing medium consisted of a 50% isopropanol and 50% ultrapure water mixture, with a flow rate of 400 µL/min. The spray voltage was adjusted to 4000 V; the sheath gas pressure was set at 30 psi; the auxiliary air flow rate was set to 10 units; the capillary temperature was set to 350 °C; the Source CID cracking collision energy was set to 0 V; and the collision pressure was set to 1.5 m Torr. The collision energy was set to −38 eV. The specified syringe volume was set to 500 µL.

To assess the intensity of photons in the incident radiation, a 7 cm long and 5 cm wide filter was selected for exposure to monochromatic visible light at a wavelength of 420 nm. The mole number of cumulative photons passing over the complete area of the filter per unit time was obtained using the formula υ = c/λ, where υ represents photonic frequency, λ denotes incident light wavelength, and c is the light velocity. The Avogadro constant (NA) and Planck constant (h) were used to calculate the energy of a photon (hv). The distance between the photoreactor and the xenon arc lamp was adjusted to regulate the incident photon flux on the photoreactor.

The incoming photon flux I_o_ was quantified through a radiometer (Model FZ-A, Photoelectric Instrument Factory Beijing Normal University, Beijing, China), and was established as 4.76 × 10^−6^ Einstein L^−1^ s^−1^ under VLTI (wavelength range of 400–700 nm). The incoming photon flux onto the photoreactor was modified by varying the distance separating the photoreactor and the Xe arc lamp.

The PNE was calculated with the following Equation (5):

*ϕ* = *R*/*I_o_* (5)

where *ϕ* is the PNE (%), and *R* is the retrogradation velocity of ENR (mol L^−1^ s^−1^), and *I_o_* is the incident photon flux (Einstein L^−1^ s^−1^).

## 4. Conclusions

In this study, a novel highly efficient photocatalyst, Sm_2_EuSbO_7_, was synthesized for the first time using the solid-state calcination method. The novel Sm_2_EuSbO_7_ photocatalytic material was pure phase with a pyrochlore structure, cubic lattice, and space group Fd3m. This was corroborated by various characterization techniques including XRD analysis, FTIR, Raman, UV-vis, XPS, and TEM-EDS. In addition, SZHP was successfully fabricated for the first time through the solvothermal method. The formation of the heterojunction between Sm_2_EuSbO_7_ and ZnBiSbO_5_ played a vital role in elevating the photocatalytic functionality, resulting in significant improvements in photocatalytic activity. The synthesized SZHP photocatalyst exhibited outstanding performance in removing pollutants, particularly PM, from wastewater. Within a duration of 150 min, the SZHP photocatalyst achieved remarkable removal rates of PM concentration and TOC concentration, reaching up to 100% and 99.45%, respectively. In comparison to Sm_2_EuSbO_7_, ZnBiSbO_5_, and N-TO, the PM removal rate of SZHP was ascertained to be 1.09 times, 1.20 times, and 2.38 times higher, respectively, demonstrating the superior performance of SZHP as a photocatalyst for PM degradation. The reinforced PCP can be linked to the efficient dissociation and diminished reassociation of light-excited charge carriers within SZHP. The heterojunction between Sm_2_EuSbO_7_ and ZnBiSbO_5_ facilitates efficient charge transfer and promotes the generation of reactive species, which enhances the degradation of PM. Additionally, this study proposed a possible degradation pathway and mechanism for PM, offering valuable insights into the photocatalytic process.

Overall, this research successfully synthesized the novel Sm_2_EuSbO_7_ photocatalyst and constructed SZHP, providing a promising approach for treating wastewater contaminated by PM. The findings contribute to the advancement of photocatalytic technology and provide valuable perspectives on the construction and enhancement of efficient photocatalysts for pollutant degradation.

## Figures and Tables

**Table 1:** Architecture dimensions of Sm_2_EuSbO_7_ fabricated by the solid-state calcination method.

Atom	x	y	z	Occupied Index
Sm	0	0	0	1
Eu	0.5	0.5	0.5	0.5
Sb	0.5	0.5	0.5	0.5
O(1)	−0.175	0.125	0.125	1
O(2)	0.125	0.125	0.125	1

**Table 2:** Architecture dimensions of ZnBiSbO_5_ fabricated by the solid-state calcination method.

Atom	x	y	z	Occupied Index
Zn	0.5	0.5	0.5	1
Bi	0	0	0	1
Sb	0.5	0.5	0.5	1
O(1)	0.125	0.125	0.125	0.5
O(2)	0.125	0.125	0.375	1

**Table 3:** Comparative analysis of the PCP of SZHP fabricated via the solvothermal method with other documented photocatalysts in the PDD of parathion methyl.

Photocatalyst	Radiation	Irradiation Time (min)	Pesticide	Removal Rate (%)	Ref.
TiO_2_	UV	300	Parathion methyl	90	[108]
ZnO/CuO	Simulated solar light	100	Parathion methyl	90	[109]
ZnO nanorod	Visible light	180	Parathion methyl	99	[2]
Bi_2_MoO_6_	Visible light	120	Parathion methyl	69	[58]
NiO/Bi_2_MoO_6_	Visible light	120	Parathion methyl	95	[58]
GO-Fe_2_O_3_	Visible light	140	Parathion methyl	40	[110]
GO-Fe_2_O_3_/Bi_2_MoO_6_	Visible light	140	Parathion methyl	98	[110]
Ag-TiO_2_	Visible light	420	Parathion methyl	100	[59]
Sm_2_EuSbO_7_	Visible light	150	Parathion methyl	92	This study
SZHP	Visible light	150	Parathion methyl	100	This study

## Data Availability

Data are contained within the article.
